# Canonical EEG microstates transitions reflect switching among BOLD resting state networks and predict fMRI signal

**DOI:** 10.1088/1741-2552/ac4595

**Published:** 2022-01-06

**Authors:** Obada Al Zoubi, Ahmad Mayeli, Masaya Misaki, Aki Tsuchiyagaito, Vadim Zotev, Tulsa 1000 Investigators, Hazem Refai, Martin Paulus, Jerzy Bodurka

**Affiliations:** 1 Laureate Institute for Brain Research, Tulsa, OK, United States of America; 2 Electrical and Computer Engineering, University of Oklahoma, Tulsa, OK, United States of America; 3 Harvard Medical School, Boston, United States of America; 4 Stephenson School of Biomedical Engineering, University of Oklahoma, Norman, OK, United States of America; 5 Deceased.

**Keywords:** EEG, blood-oxygenation-level dependent, resting state, fMRI, machine learning, EEG microstate

## Abstract

**Objective.:**

Electroencephalography (EEG) microstates (MSs), which reflect a large topographical representation of coherent electrophysiological brain activity, are widely adopted to study cognitive processes mechanisms and aberrant alterations in brain disorders. MS topographies are quasi-stable lasting between 60–120 ms. Some evidence suggests that MS are the electrophysiological signature of resting-state networks (RSNs). However, the spatial and functional interpretation of MS and their association with functional magnetic resonance imaging (fMRI) remains unclear.

**Approach.:**

In a cohort of healthy subjects (*n* = 52), we conducted several statistical and machine learning (ML) approaches analyses on the association among MS spatio-temporal dynamics and the blood-oxygenation-level dependent (BOLD) simultaneous EEG-fMRI data using statistical and ML approaches.

**Main results.:**

Our results using a generalized linear model showed that MS transitions were largely and negatively associated with BOLD signals in the somatomotor, visual, dorsal attention, and ventral attention fMRI networks with limited association within the default mode network. Additionally, a novel recurrent neural network (RNN) confirmed the association between MS transitioning and fMRI signal while revealing that MS dynamics can model BOLD signals and vice versa.

**Significance.:**

Results suggest that MS transitions may represent the deactivation of fMRI RSNs and provide evidence that both modalities measure common aspects of undergoing brain neuronal activities. These results may help to better understand the electrophysiological interpretation of MS.

## 1. Introduction

Electroencephalography (EEG) is a direct measure of brain neuronal activity with a high temporal resolution (e.g. order of milliseconds), although it suffers from low spatial resolution. Distinct topographic representations of the EEG electric scalp potentials—lasting a few dozens of a millisecond, termed EEG-microstates (MSs)—provide a novel opportunity to find potential biomarkers of various psychiatric disorders [1]. Understanding the spatial and functional brain representation of MS is an active field of research. One accepted notion is that MS may represent the spatial and temporal neuronal current activity [1, 2]. Thus, a change in the topographies of MS may be attributed to a change in the orientation or distribution of the current dipoles underlying EEG signal formation [3, 4]. In addition, MS are correlated with several mental processes, including attention, language processing, perceptual awareness, various resting-state networks (RSNs), and visual processing [2, 5-12]. Furthermore, MS has been used to characterize different mental disorders such as schizophrenia, Alzheimer’s disease, dementia, mood or anxiety disorders, and autism [13-28].

MS has high temporal resolution; however, it suffers from low spatial resolution. Thus, several studies have investigated the association between MS [3, 29, 30] and functional magnetic resonance imaging (fMRI) signals, revealing a correlation between the MS time course and fMRI RSNs [10, 29, 31]. Moreover, the source localization of MS was investigated in a previous study [32], where authors identified seven MS (A through G) and localized their sources from healthy volunteers. Their results suggested a common fMRI activation among MS in several brain hubs, e.g. anterior and posterior cingulate cortices, insula, superior frontal cortex, and precuneus. Other works have used the voxel-wise generalized linear model (GLM) analysis to associate MS time courses with blood-oxygen-level-dependent (BOLD) signals. For instance, using canonical MS analysis [2], the authors in [10] demonstrated that for resting-state fMRI: (a) MS A (MS-A) is negatively correlated with the BOLD signal in the bilateral superior and middle temporal lobe; (b) MS B (MS-B) is negatively correlated with the BOLD signal in the bilateral occipital cortex; (c) MS C (MS-C) is positively correlated with the BOLD signal in the right insular cortex, bilateral inferior frontal cortices, and the dorsal anterior cingulate cortex; and (d) MS D (MS-D) is negatively associated with BOLD signal within frontoparietal regions. Similarly, authors [31] extracted 10 MS from healthy volunteers, revealing a significant association between the spatial maps of MS and BOLD signals in multiple brain regions. Using frequency-varying MS information, the authors in [33] found that thalamic nuclei subregions exhibit different BOLD fMRI activation patterns based on the employed frequency.

The previous EEG-fMRI results suggested that MS time courses are associated with one or more brain RSNs activities, and the brain network activities sum up and contribute to a specific MS class. However, this assumption was recently reconsidered by studying the impact of the intracortical sources of EEG alpha oscillation on the topography of the MS classes [34]. The results revealed that the intracortical strength of alpha oscillation and its distribution predominantly determined the MS topographies. Additionally, the authors in [35] showed that MS occurrence, coverage, and duration are influenced by EEG alpha oscillation and are sensitive to intra- and inter-subject alpha rhythm variability, further indicating a prominent influence of brain oscillation on the MS. The alpha rhythm was shown to decrease during task-related mental activity [36-39]. Additionally, alpha rhythm has been shown to have a modularity effect on the BOLD signal [36, 40], while exhibiting an anticorrelation relationship epically for the occipital cortex, observed in fMRI and other imaging modalities [39, 41-44]. In addition, based on findings showing that (a) alpha power modulates MS [34, 35]; (b) alpha power associated with BOLD signals; and (c) the consistent association of BOLD signal and alpha rhythm, we proposed and posited the notion that MS represent transitions among BOLD RSNs rather than an MS represents specific BOLD RSN.

We, therefore, investigated the spatial maps of the MS direct time courses, activity, and pairwise transitions by utilizing and conducting GLM analysis. More specifically, we extracted the canonical MS classes for the group level of subjects. Then we extracted the following information: (a) direct time courses of the four canonical MS (A through D) for each subject (4 × MSs regressors); (b) the activity per each of the MS classes); and (c) pairwise transitions among the four MS classes. The information was then used as regressors for GLM analysis (activity: 4×MSs regressors; transitions: 4×MSs×4×pairwise transitions regressors). For each regressor, we generated the corresponding fMRI correlated maps (fCMs) and quantified the spatial overlap with other RSNs.

In addition to the GLM analysis, we introduced a machine learning (ML) approach using a novel multi-output time-delay neural network (mTDNN) to assess the amount of information between MS and BOLD signals. Single output TDNN has been used widely to study problems with temporal dependencies [45-48]. Recently, several efforts have been made to model EEG and fMRI and avoid the need for high-cost fMRI recordings [49, 50]. Our modeling benefits from the fact that MS are broadband and multichannel representations and hence have less complexity as compared to other EEG features. Thus, we harnessed mTDNN to model MS and BOLD signals and reveal the amount of temporal relationship between MS information and BOLD signals estimated from Yeo’s 7-network atlas [51].

## 2. Method

### 2.1. Participants

The data from 52 healthy control subjects (mean age: 32, standard deviation (SD): 12 years; 25 females) participated in the Tulsa 1000 (T-1000) study (the first 500 subjects) were used for this study. Tulsa 1000 is a naturalistic study assessing and longitudinally following 1000 individuals, including healthy individuals and treatment-seeking individuals with substance use, eating, mood, and/or anxiety disorders [52]. Simultaneous EEG-fMRI recording was obtained from each subject during a resting-state scan. The study was conducted with a research protocol approved by the Western Institutional Review Board. All volunteers provided written informed consent and received financial compensation for their time to participate in this study. The participants were asked to relax and keep their eyes open and fixate their eyes on a cross displayed on the fMRI stimulus projection screen. Further quality control for the simultaneous EEG-fMRI data (see below) reduced the number of subjects to 40 participants (age mean ±SD: 32 ± 12 years; 23 females).

### 2.2. Simultaneous EEG-fMRI data acquisition

MRI imaging and simultaneous EEG-fMRI were conducted on a General Electric Discovery MR750 whole-body 3 T MRI scanner with a standard 8-channel, receive-only head coil array. A single-shot gradient-recalled echoplanar imaging (EPI) sequence with sensitivity encoding (SENSE) was acquired with the following parameters: TR = 2000 ms, TE = 27 ms, FA = 78°, FOV = 240 mm, 39 axial slices with 2.9 mm thickness with 0.5 mm gap, matrix = 96×96, SENSE acceleration factor *R* = 2. EPI images were reconstructed into a 128 × 128 matrix that produced 1.875 × 1.875 × 2.9 mm^3^ voxel volume. The resting fMRI run time was 8 min 8 s (244 volumes). To provide anatomical reference, T1-weighted MRI images were acquired using a magnetization-prepared rapid gradient-echo (MPRAGE) sequence with the following parameters: FOV = 240 × 240 mm, matrix = 256 × 256, 120 axial slices, slice thickness = 0.9 mm, 0.938 × 0.938 × 0.9 mm^3^ voxel volume, TR = 5 ms, TE = 1.948 ms, *R* = 2, flip angle = 8°, delay time = 1400 ms, inversion time = 725 ms, sampling bandwidth = 31.2 kHz, scan time = 5 min 4 s. During each scan, a pneumatic belt placed around the subject’s torso was used to record respiration, and a photoplethysmograph with an infrared emitter placed under the pad of the subject’s index finger was used to record pulse oximetry. The resting-state fMRI scan was the first scan of each session. During each resting-state scan, subjects were instructed to remain still with their eyes open while looking at a fixation cross on the screen. The only other instruction provided was to ‘try to clear your mind and do not think about anything in particular.’

EEG signals were recorded simultaneously with fMRI using a 32-channel, MR-compatible EEG system (Brain Products GmbH) with electrodes arranged according to the international 10–20 system. Electrocardiogram signal was recorded using an electrode on the subject’s back. In order to synchronize the EEG system clock with the 10 MHz MRI scanner clock, a Brain Products SyncBox device was utilized. EEG temporal and measurement resolutions were 0.2 ms (i.e. 16-bit 5 kS s^−1^ sampling) and 0.1 *μ*V, respectively. Hardware filtering throughout acquisition in a frequency band between 0.016 and 250 Hz was applied to EEG signals.

### 2.3. EEG data preprocessing

The following preprocessing steps were performed in BrainVision Analyzer 2 software, as described in [53]. More specifically, MRI imaging artifacts within the EEG signal were reduced using the average artifact subtraction (AAS) method [54]. After down-sampling EEG signals to 250 Hz, band-rejection filters with 1 Hz bandwidth [55-57] were applied to reduce fMRI slice selection fundamental frequency (19.5 Hz) and its harmonics, mechanical vibration noise (26 Hz) along with an alternating current power line noise (60 Hz). Another bandpass filter from 0.1 to 80 Hz (48 dB/octave) was applied to the EEG data. Ballistocardiogram (BCG) artifacts also were reduced using AAS [58-60]. EEG was then decomposed to neural activity and artifactual independent components (ICs) using the independent component analysis (ICA) Infomax algorithm [61] implemented in Analyzer 2. Manual inspection of ICs properties was used to determine artifactual ICs. We used power spectrum density, topographic maps, time course signals, kurtosis values, and energy values for detecting and removing artifactual ICs, including imaging and residual BCG, muscle, and ocular artifacts. Finally, a back-projection (i.e. inverse ICA) was applied after selecting ICs related to neural activities.

### 2.4. MS analysis

The standard spatially independent MS analysis described in [2] was conducted. EEG was referenced using an average [2]. The number of desired MS was set to *k* = 4. The following steps were required before running the clustering algorithm: first, the global field power (GFP) for each subject was calculated from band-passed filtered EEG data between 2 and 20 Hz [2, 18]. GFP peaks were then identified after smoothing the data with a Gaussian-weighted moving average of 5 samples. Finally, to offer a higher level of accuracy, we randomly selected up to *n* = 10 000 peaks and extracted the corresponding EEG points for later analysis. The selected EEG points were then submitted to the atomize and agglomerate hierarchical clustering algorithm [62, 63] to identify four MS templates. Next, the group means of MS were computed by first sorting individual MS and then finding the common topography across all subjects. It should be noted that some investigators have used 1–40 Hz filtering [2] instead of 2–20 Hz to delineate MS. Thus, we repeated MS analysis using 1–40 Hz filtering (and subsequent analyses) to test the effect of EEG filtering on the association with BOLD signals. All the corresponding results of 1–40 Hz filtering are presented in the supplement.

### 2.5. Extracting MS regressors

**Figure 1.**
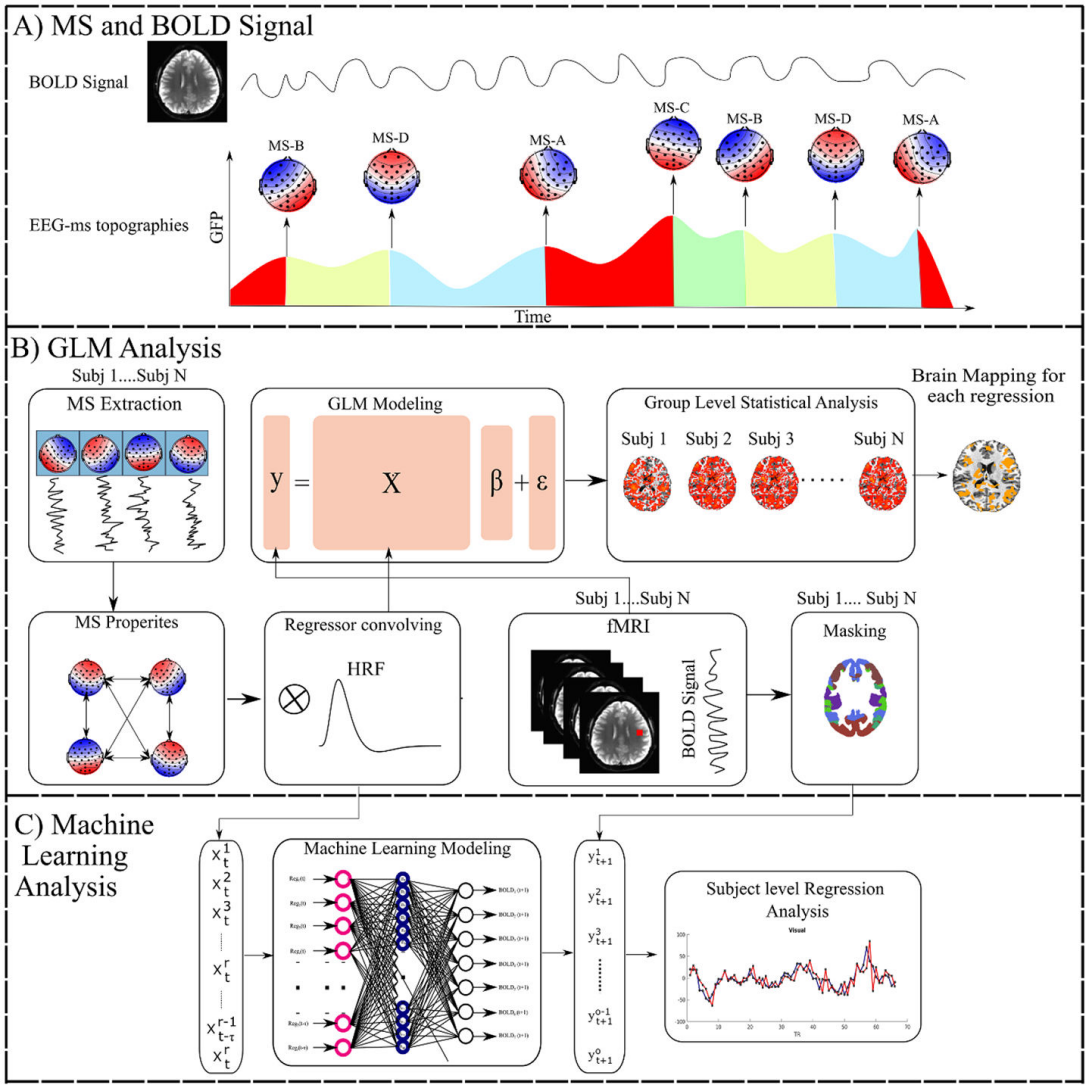
The analyses pipelines overview of investigating the MS associations with BOLD fMRI signals. Figure (A) shows canonical MS (A through D) temporal changes and the corresponding changes in the BOLD signal from the voxel. Figure (B) depicts the statistical analysis pipeline to identify the spatial maps of MS direct time courses, activity, and transitions information using GLM. Figure (C) adopts a novel ML to reveal the BOLD signals and MS associations.

First, we revisited MS modeling and extraction to elaborate on the extracted MS regressors. Canonical MS analysis relies on segmenting EEG points according to their spatial similarities. Although EEG points can be selected randomly, GFP is commonly used to select potential EEG points for segmentation. GFP is defined as the spatial standard deviation of EEG signals across all channels. It has been shown that the peaks of GFP maintain a high signal-to-noise ratio [1, 3]. Thus, focusing on GFP peaks to select potential EEG points for segmentation may improve results (see figure 1). Furthermore, using GFP peaks helps with reducing the dimensionality of EEG data

(1) $$\text{GFP}=\sqrt{\frac{\sum_{i=1}^{p}{\left(\nu_{i}(t)-{\bar{\nu }}_{i}(t)\right)}^{2}}{p}}$$

where p is the number of electrodes; ${\bar{\nu }}_{i}(t)$ is the mean of electrode values at time point t; and $\nu_{i}(t)$ is the value of electrode i at time point t.

In this work, we constructed and employed three types of regressors for EEG-informed fMRI analysis: (a) the direct time courses of MS A through D; (b) activity per MS, which measures the extent of switching out of each MS; and (c) the transition activity between each pair of MS. It should be noted that the term *time course* here implies a different meaning from other methods that involve time course extraction, such as ICA. MS time course reflects the spatial similarity between each MS template (MS-A, MS-B, MS-C, and MS-D) and topographical representation of EEG points. Another difference that arises with the definition of the MS time course is the polarity consideration of MS, wherein different interpretations can be drawn if polarity was to be considered.

To provide a better understanding of the time course of MS, the following section describes the mathematical representation of MS regressors. First, let us consider $x_{t}$ to be the electrodes’ value at the time t and n is the total number of samples. Using subscript indexing for time and superscript indexing for MS, the MS analysis assumes that each EEG point can be presented as:

(2) $$x_{t}=\sum_{i=1}^{K}a_{t}^{i}\times {\text{MS}}^{i}+\in_{t}\;\;t\in [1,n]$$

where $x_{t}$ is the electrodes’ value vector at time t; $a_{t}^{i}$ is a factor related to ${\text{MS}}^{i}$ at each time point; and $\in_{t}$ is the error term associated with assigning that time point to one particular ${MS}^{i}$ (i.e. noise due to the lack of explained topographical representation of that point by the assigned MSs template K is the number of the assumed MS patterns). ${\text{MS}}^{i}$ is 1×p vector representing the template of ${\text{MS}}^{i}$. Typically, ${\text{MS}}^{i}$ can be one of four canonical MSs (A through D) identified in the literature [2]. It should be noted that $\|{\text{MS}}^{i}\|=\|x_{t}\|=1$ and thus the cosine similarity is equivalent to the dot product. The entire EEG recoding can be represented as matrix x=p×n with p is the number of electrodes and n is length of recordings.

The direct time course of ${\text{MS}}^{i}$ (${\text{DT}}^{i}$) can be defined as the cosine similarity among each EEG point and each ${\text{MS}}^{i}$ template as follows:

(3) $${\text{DT}}^{i}=\text{abs}\left(x^{T}\times {\text{MS}}^{i}\right).$$

This result is a vector with n×1 values between [0, 1]. The absolute term in the equation accounts for the polarity invariant property of MS analysis.

In addition to the direct time course regressors, the activity of each MS was extracted using a sliding window. More specifically, the activity metric measures the switches over time from one microstate to another ones. To extract activity regressors, we need to assign each EEG point to one of the K MSs based on the similarity values. First, let us assume MS are represented by matrix of p×K, where K is the number of MSs and p is the number of electrodes. The similarity f among each EEG point and the K MSs can be represented as:

(4) $$f=\text{abs}\left(x^{T}\times \text{MS}\right)\;\;=\left[\begin{array}{ccccc} f_{1}^{1} & f_{1}^{2} & \cdots & f_{1}^{K-1} & f_{1}^{K}\\ f_{2}^{1} & f_{2}^{2} & \cdots & f_{2}^{K-1} & f_{2}^{K}\\ \vdots & \; & \ddots & \; & \vdots \\ f_{n-1}^{1} & f_{n-1}^{2} & \cdots & f_{n-1}^{K-1} & f_{n-1}^{K}\\ f_{n}^{1} & f_{n}^{2} & \cdots & f_{n}^{K-1} & f_{n}^{K} \end{array}\right].$$

This results in matrix n×K, with $f_{t}^{i}$ represents similarity between EEG at index t with ${\text{MS}}^{i}$. In order to assign each EEG to one MS, we used winner-take-all, i.e. EEG points are assigned to the microstate with the highest similarity as follows:

(5) $$L=\underset{K}{\text{argmax}}(f)=\left[\;\begin{array}{c} L_{1}\\ L_{2}\\ \vdots \\ L_{n-1}\\ L_{n} \end{array}\;\right].$$

L(n×1) are the assigned labels for each EEG point. $L_{t}$ can take a value from [1, K], corresponding to one of the K MSs. For example, if L=[1,1,1,3,3,4,4,…], then EEG points at that indices 1, 2 and 3 were assigned to MS^1^; indices 3 and 4 were assigned to MS^3^; indices 5 and 6 were assigned to MS^4^. As aforementioned, MS^1^ can be one of the four canonical MSs referred to them conventional as A through D.

MS activity regressor of ${\text{MS}}^{i}$ (${\text{AT}}_{w}^{i}$) is calculated as the summation of the number of switches from microstate ${\text{MS}}^{i}$ to other MSs within a window size w=2 s. To obtain the regressors for the entire duration (n points), the window was shifted by one sample, and the metric was recalculated again. Mathematically ${\text{AT}}_{w}^{i}$ can be written as:

(6) $$\;{\text{AT}}_{w}^{i}=\sum_{w-1}^{t=1}(L_{t+1}^{j}|L_{t}^{i})\;\text{with}\;i\neq j;i,j\in [1,K];\;\text{and}\;t\in [1,w]\;.$$

With $L_{t}^{i}$ is the label of ${\text{MS}}^{i}$ at index t and $L_{t+1}^{j}$ is label of ${\text{MS}}^{j}$ at the index t+1. For instance, if L=[1,1,1,3,3,4,4,1,1,2,2,1,2,3], then AT^1^ = 3.

We also calculated the transition regressor ${\text{TS}}_{w}^{i\to j}$ among pairs of MSs. Similar to activity regressors, we used a window size w=2 s to estimate the number of transitions between paris of MSs. ${\text{TS}}_{w}^{i\to j}$ can be written as follows:

(7) $$\;{\text{TS}}^{i\to j}=\sum_{w-1}^{t=1}(L_{t+1}^{j}|L_{t}^{i}))\;\text{with}\;i,j\in [1,K];\;\text{and}\;t\in [1,w]\;.$$

**Figure 2.**
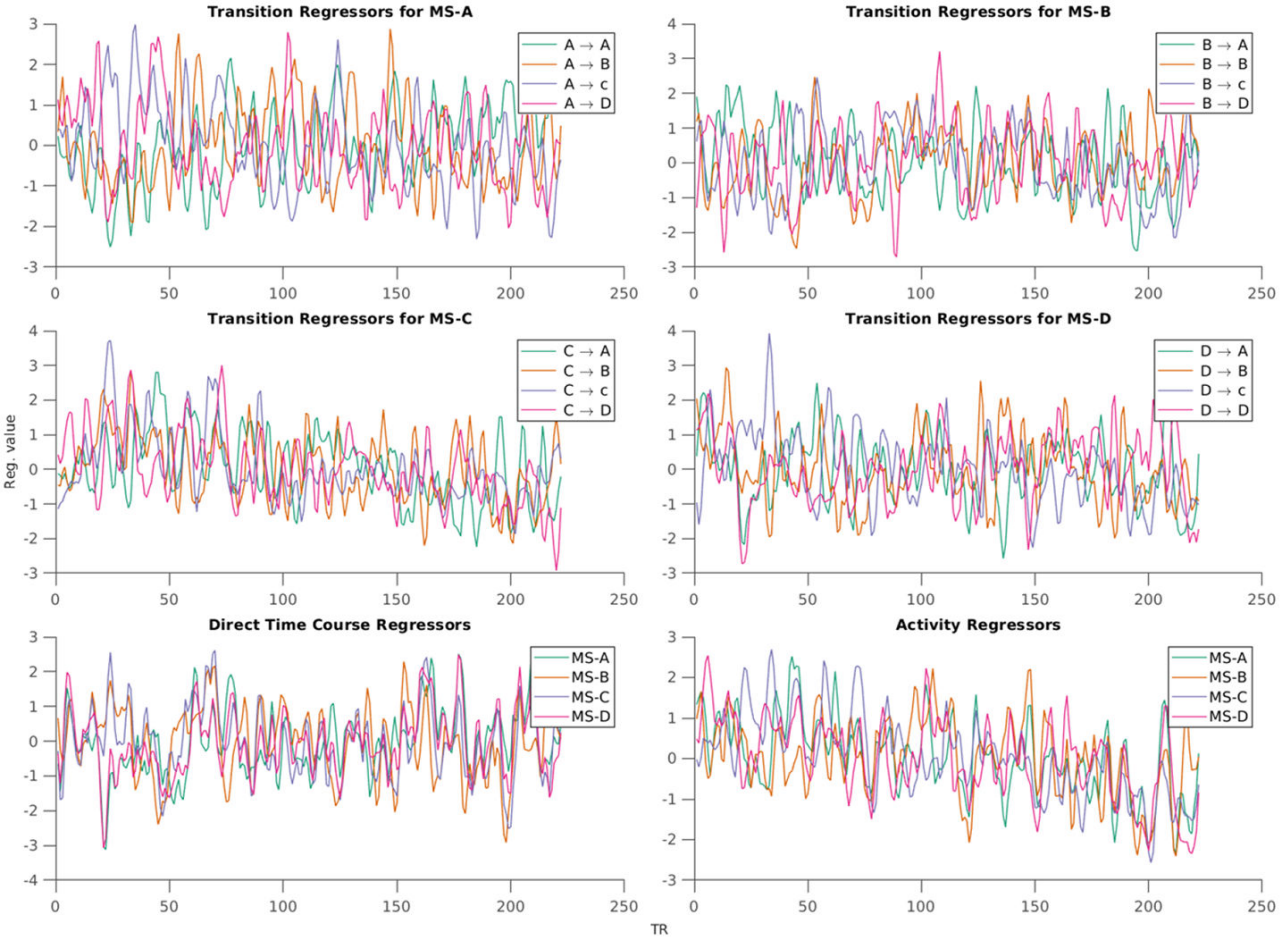
An example of transition, activity, and direct time courses regressors from exemplar subject.

Finally, each regressor was convolved with double-gamma hemodynamic response function [64], standardized, and then down-sampled to the resting state repetition time (TR) of fMRI, resulting in MS-informed regressors to be included in BOLD signal analysis. Figure 2 shows an example of the resulting regressors.

### 2.6. GLM analysis

Imaging analyses were carried out using the Analysis of Functional NeuroImages (AFNI) software (http://afni.nimh.nih.gov/afni/). The afni_proc.py command was employed to preprocess the data using the default parameters unless otherwise noted. The first three volumes were omitted from the analysis to discard non-steady state BOLD signals. The despike option was applied to avoid sudden jumps in the time courses of BOLD signals. RETROICOR [65] and respiration volume per time correction [66] was applied to remove cardiac- and respiration-induced noise in the BOLD signal. Slice-timing differences were adjusted by aligning to the first slice, and the volumes were aligned to a base image using the 3dvolreg AFNI program with two-pass registration. The volume with the minimum outlier fraction of the short EPI dataset acquired immediately after the high-resolution anatomical (MPRAGE) brain image was used as the registration base. The anatomical image was aligned to the base EPI image, and the aligned anatomical image was warped into the Montreal Neurological Institute template brain using an affine transformation (@auto_tlrc AFNI program). The warping to the template with resampling to 2 mm^3^ voxels was applied to the functional volumes. Data were then smoothed using a Gaussian kernel of 6 mm FWHM and then scaled to have a mean of 100 and a range of [0, 200].

fMRI BOLD signal analysis was performed using the standard GLM approach with the AFNI 3dDeconvolve function [67]. The design matrix included one MS regressor corresponding to one MS property (direct time, activity, or transition) and a set of nuisance covariates, including: (a) low-frequency fluctuation from the signal time course (i.e. 3rd-order polynomial model); (b) 12 motion parameters (i.e. three-shift and three rotation parameters with their temporal derivatives); (c) three PCs of the ventricle signal from the signal time course; and (d) local white matter average signal (ANATICOR) [68]. The ventricle and the white matter masks were derived from FreeSurfer segmentation of the anatomical image. GLM β coefficients were computed for each voxel, and then a one-sample t-test was applied to the entire sample of healthy subjects to extract MS templates. To control for potential false positives error in BOLD signal [69], (a) the non-Gaussian spatial autocorrelation function (ACF) was estimated for the dataset; (b) AFNI’s 3dClustSim was applied to the statistical map [70]; (c) a permutation test (n=10000) was performed using the Smith procedure [71], showing that an ACF-corrected cluster requires a minimum of 136 voxels to be deemed significant at *p* < 0.05—using an uncorrected voxel-wise threshold of *p* < 0.005. Moreover, the GLM model excluded TRs with severe motion (root mean square > 0.2) or with EEG artifact (i.e. if the TR was located within unusable intervals of EEG data). In addition to the previous steps, further exclusion criterion was applied to subjects’ fMRI datasets, given that the number of censored volumes. In details, we excluded any subject if the number of censored TRs was more than a third of the total number of TRs. This was necessary to ensure that the GLM model had enough data to estimate beta coefficients.

### 2.7. ML for modeling MS and BOLD signals

#### 2.7.1. Input and output

**Figure 3.**
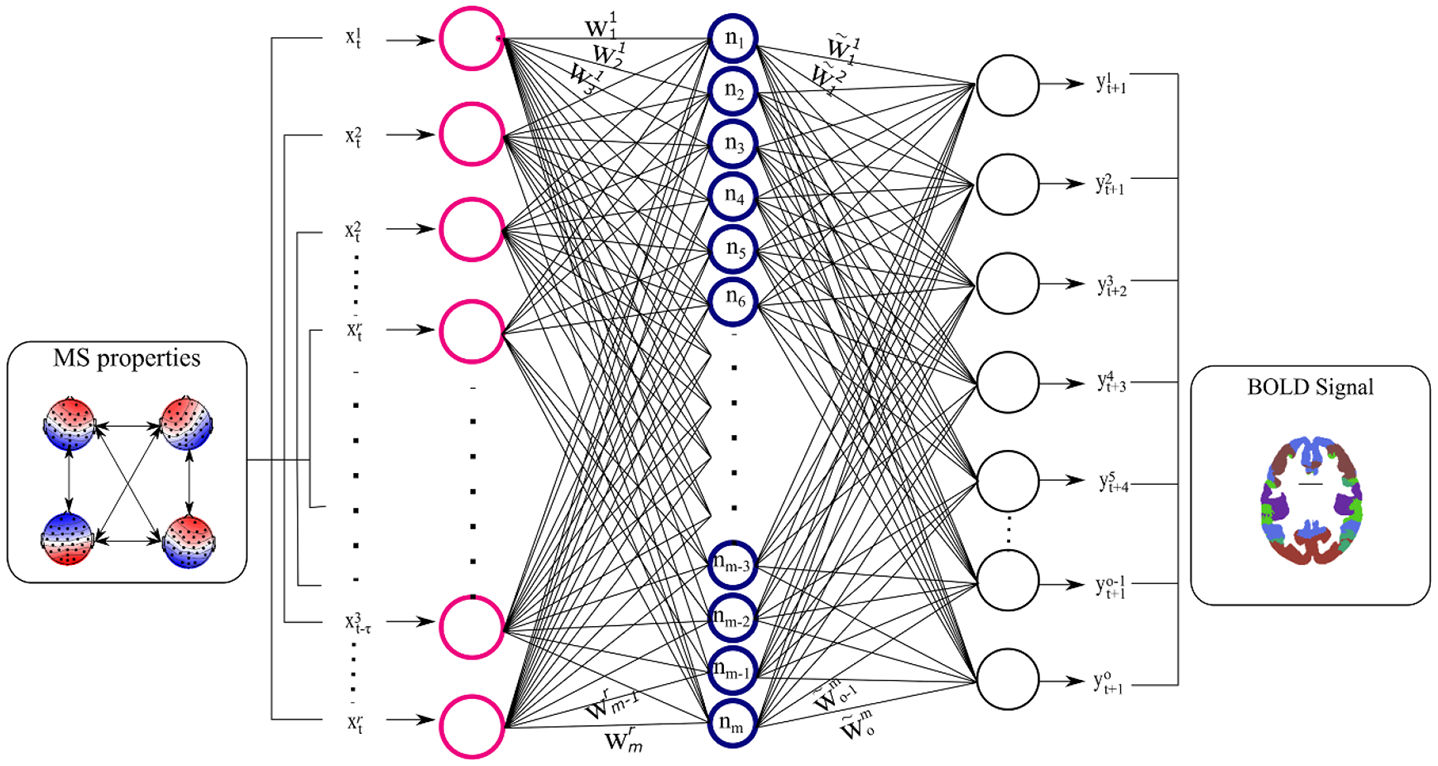
Multi-output time-delay neural network for modeling BOLD signals from MS properties for one step ahead prediction. A similar Multi-output time-delay neural network was also used to predict MS properties from BOLD signals. Overall, four experiments were conducted: (a) predicting BOLD signals from the MS dynamics (X=BOLD signals; o=7; Y=MS dynamics; r=20); (b) predicting BOLD signals from MS time courses (X=BOLD signals; o=7; Y=MS time courses; r=4); (c) predicting MS dynamics from the BOLD signals (X=MS dynamics; o=20; Y=BOLD signals; r=7); and (d) predicting MS time course from the BOLD signals (X=MS time courses; o=4; Y=BOLD signals; r=7).

To reveal the association between MS information and BOLD signals, we adopted a novel mTDNN architecture. The proposed architecture uses shared weights across the outputs (BOLD signals from the brain), such that the neural network will learn the overall relationship between inputs and brain networks. mTDNN offers sequence modeling of the past inputs to predict the information at the current TR. To reduce the complexity of the problem, we extracted the BOLD signal using the 7-network parcellation from Yeo’s atlas [51]. We conducted overall four experiments to find the amount of information that MS direct time (DT) and MS dynamics (activity [AT] and transition [TS]) about the BOLD signal and vice versa. Each experiment used either the MS direct time course regressors as input ([DTMSA,DTMSB,DTMSC,DTMSD]), number of inputs (r=4), or MS dynamics $\left(\left[{\text{AT}}^{{\text{MS}}^{A}},{\text{AT}}^{{\text{MS}}^{B}},{\text{AT}}^{{\text{MS}}^{C}},{\text{AT}}^{{\text{MS}}^{D}},{\text{TS}}^{{\text{MS}}^{A}\to {\text{MS}}^{A}},{\text{TS}}^{{\text{MS}}^{A}\to {\text{MS}}^{B}},\ldots ,{\text{TS}}^{{\text{MS}}^{D}\to {\text{MS}}^{D}}\right],r=20\right)$. Each time, we assessed the correlation between the true and the predicted BOLD signals (number of outputs o=7) for one step and two steps in the future (t+1 and t+2). Figure 3 depicts the proposed architecture of mTDNN modeling with inputs represented as $x_{t-\tau }^{r}$, where r is input index, o is the output index, and t−τ is the time delay at which regressor is measured. Equations (8) and (9) describes the optimization process to find the shared weights W and $\tilde{W}$:

(8) $$Z=W\times X=\left[\;\begin{array}{c} z^{1}\\ z^{2}\\ \vdots \\ z^{m-1}\\ z^{m} \end{array}\;\right]\;\;=\left[\begin{array}{ccc} w_{1}^{1} & \cdots & w_{1}^{r}\\ w_{1}^{1} & \cdots & w_{1}^{r}\\ \vdots & \ddots & \vdots \\ w_{m-1}^{1} & \cdots & w_{m-1}^{r}\\ w_{1}^{m} & \cdots & w_{m}^{r} \end{array}\right]\times \left[\;\begin{array}{c} x_{t}^{1}\\ x_{t}^{2}\\ \vdots \\ x_{t-\tau }^{r-1}\\ x_{t-\tau }^{r} \end{array}\;\right]$$

(9) $$Y=\tilde{W}\times Z=\left[\;\begin{array}{c} y_{t+1}^{1}\\ y_{t+1}^{2}\\ \vdots \\ y_{t+1}^{o-1}\\ y_{t+1}^{o} \end{array}\;\right]\;\;=\left[\begin{array}{ccc} {\tilde{w}}_{1}^{1} & \cdots & {\tilde{w}}_{1}^{m}\\ {\tilde{w}}_{2}^{1} & \cdots & {\tilde{w}}_{2}^{m}\\ \vdots & \ddots & \vdots \\ {\tilde{w}}_{6}^{1} & \cdots & {\tilde{w}}_{6}^{m}\\ {\tilde{w}}_{7}^{1} & \cdots & {\tilde{w}}_{2}^{m} \end{array}\right]\times \left[\;\begin{array}{c} z^{1}\\ z^{2}\\ \vdots \\ z^{m-1}\\ z^{m} \end{array}\;\right]$$

In addition to predicting BOLD signal from MS properties, we also conducted two other experiments to predict MS properties (o=20 for MS dynamics and o=4 for MS direct timecourses) from BOLD signals (r=7). This allows for a better characterization of the mutual information between MS and BOLD signals.

#### 2.7.2. Training and validation

To train mTDNN, we used the Bayesian regularization backpropagation algorithm implemented in MATLAB with 100 epochs. To find the optimal architecture in terms of the number of neurons and delays, we utilized a grid search on the first 30 TRs only. The grid search procedure aimed at finding the best number of neurons in the hidden layer n=[5,10,20,40] and the number of past TRs to take into consideration t=[1,2,3,4,5]. The model with the highest average correlation between true and the predicted outputs was selected for building the subject-level models. We ran an independent grid search for each of the four experiments; (a) predicting BOLD signals from the MS dynamics; (b) predicting BOLD signals from MSs time courses; (c) predicting MSs dynamics from the BOLD signals; and (d) predicting MSs time course from the BOLD signals. Please see the results of the grid search in figure S1 available online at stacks.iop.org/JNE/18/066051/mmedia of supplements. To prevent biasing the results, we evaluated the performance of each experiment using a time series cross-validation approach with an initial length of 31 TRs (figure S2 in supplement depicts the time series validation procedure). More specifically, we used the first 31 TRs for training and tested predicting two TRs ahead. Then, we expanded our training window to include TR at index 32 and tested predicting two TRs ahead. We repeated the process of expansion and testing until testing the last TR. The process of evaluation was done subject by subject. The results were reported as the average Pearson’s correlation between true and predicted outputs for one TR ahead prediction and two TR ahead predictions.

## 3. Results

### 3.1. MS identification

**Figure 4.**
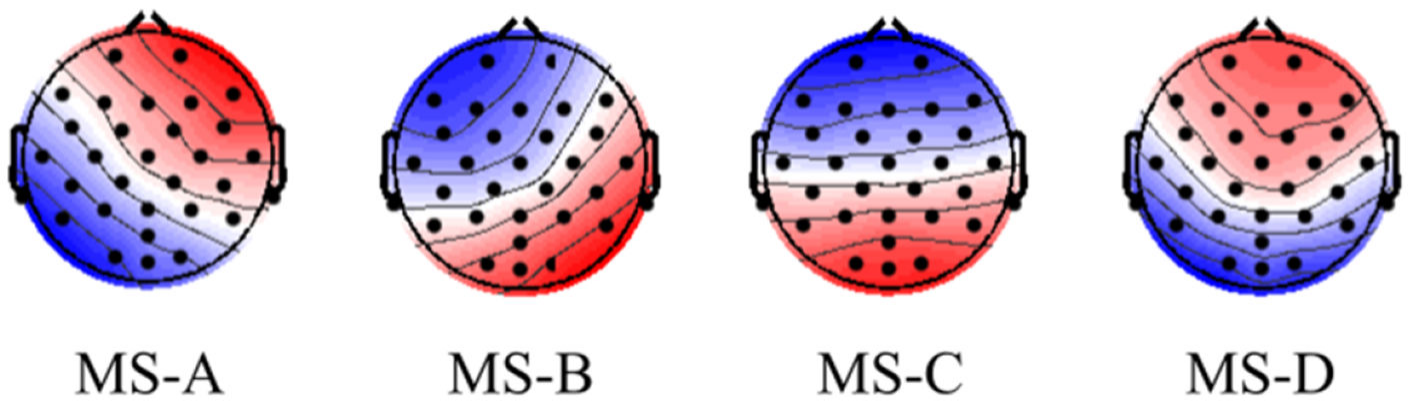
MS topographies obtained from our EEG dataset using [2–20] Hz filtering.

Using our EEG datasets, we identified the four canonical MS, which replicates MS templates obtained in the literature [2]. The raw explained variance by the four MS was 73%, with 21%, 16%, 20%, and 16% for MS-A, MS-B, MS-C, and MS-D, respectively. Figure 4 shows the extracted templates. The extracted canonical MS were then used to extract subject-level information for applying GLM and ML analyses.

Using equation (3), we extracted the direct MS regressors. Due to the high correlation among those regressors (range = [−0.6, 0.95]), each regressor was used independently in the GLM model.

### 3.2. Spatial maps of MS using direct time course regressors

**Figure 5.**
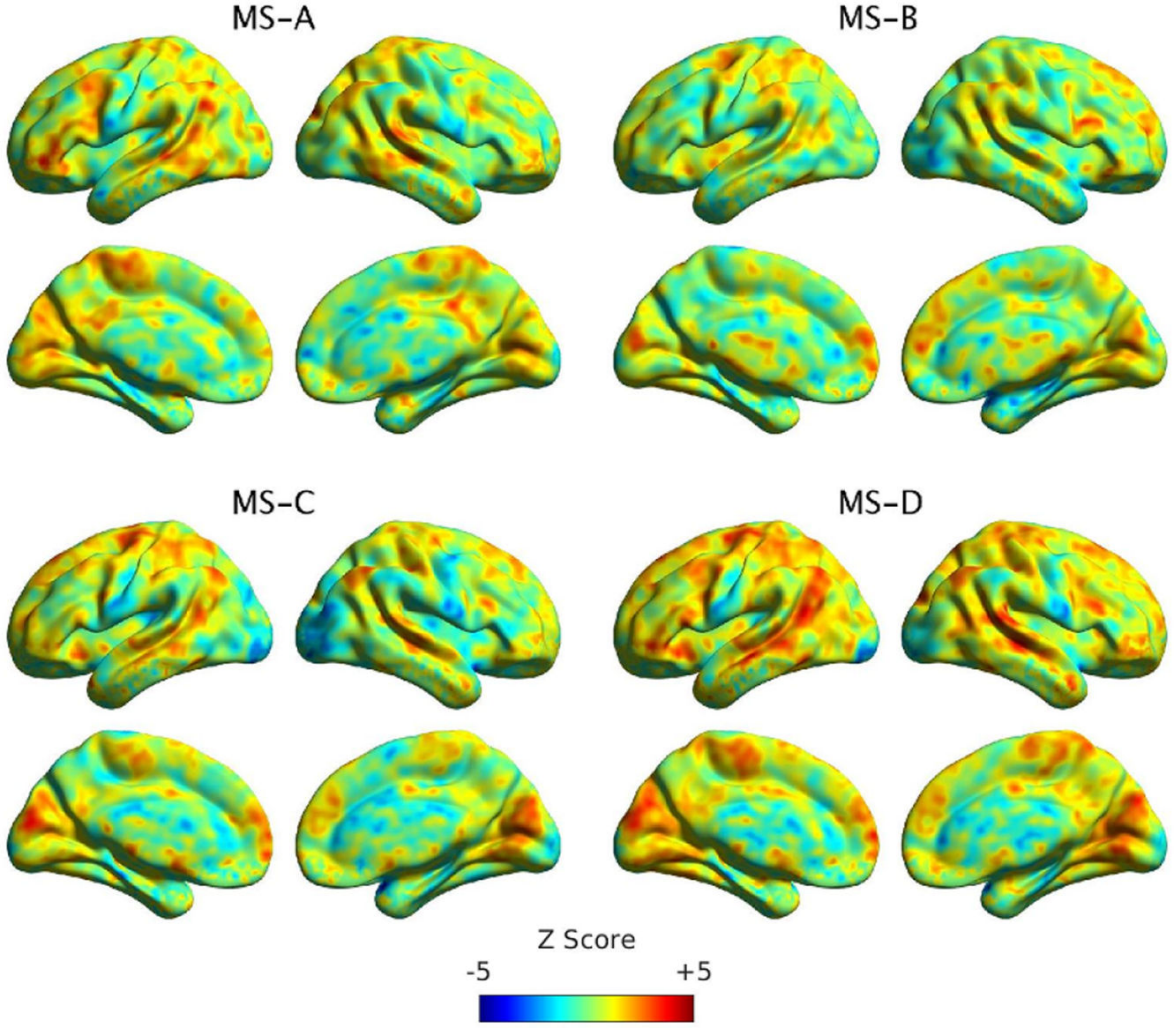
The un-thresholded maps of each MS direct time regressor. No significant clusters were found for MS-B (please refer to figure S3 in the supplement for thresholded clusters). Information about each cluster and the corresponding brain regions is shown in table 1.

MS templates were used to extract the direct time regressors of MS A through D using equation (3). Then, we derived the fCMs of each regressor using GLM analysis described before. Figure 5 shows the un-thresholded fCMs of each MS and figure S3 in the supplement shows the thresholded fCMs maps. Only nine clusters passed clustering correction. Table 1 shows the location of each cluster and the name of brain regions with the number of voxels within each cluster. We also used [1–40] Hz filtering to estimate the fCMs for direct time regressors. The un-thresholded and thresholded for [1–40] Hz filtering are shown in figures S4 and S5, respectively. The results indicate limited associations among direct time course regressor and BOLD signals, regardless of the utilized EEG filtering. Thus, we investigated the fCMs of activity and transition regressors.

### 3.3. Spatial maps of MS activity regressors

**Figure 6.**
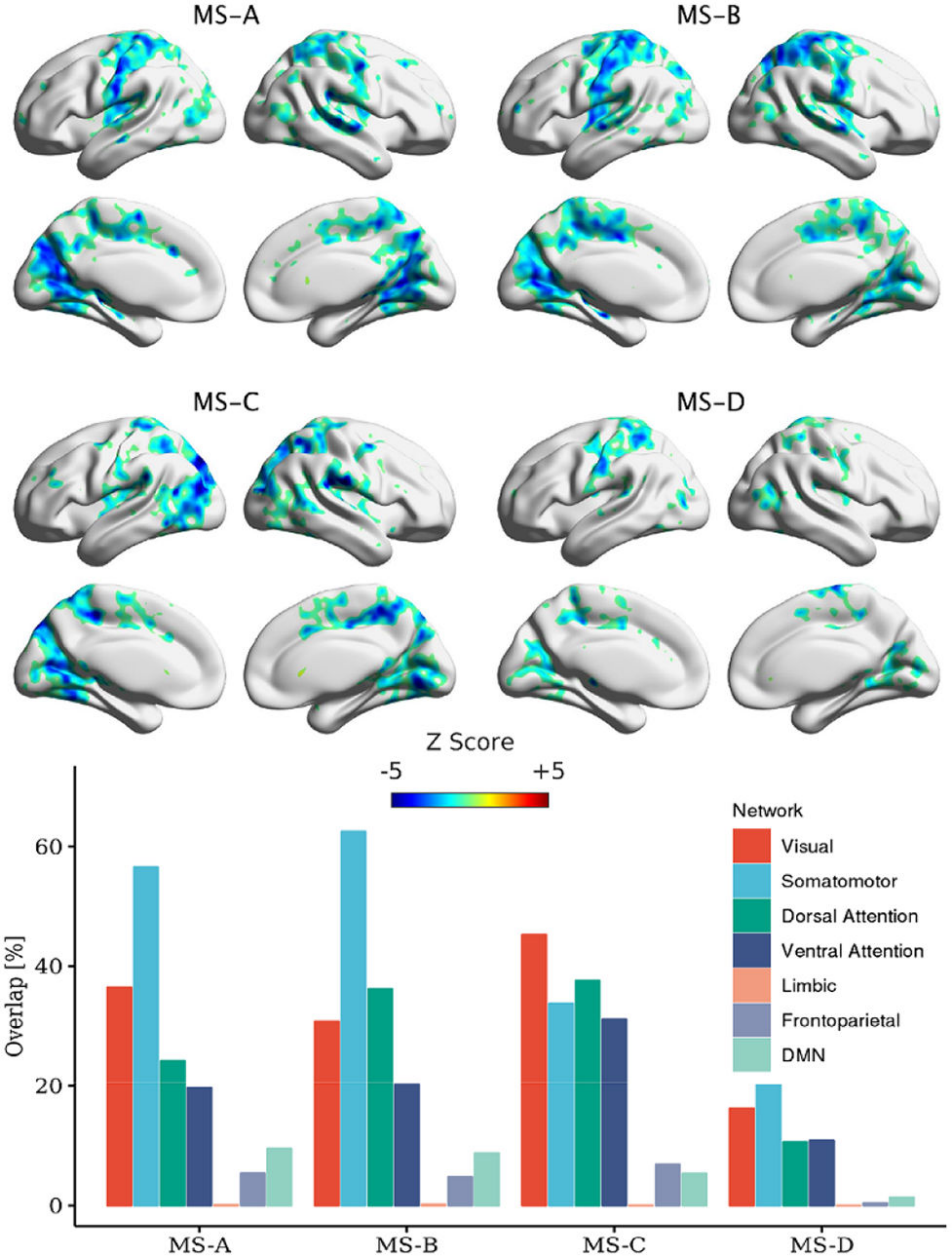
Significant clusters for MS-A, MS-B, MS-C, and MS-D using activity regressors. Clustering was performed at *p* < 0.005 and corrected at *p* < 0.05. The percentage of overlap with each of Yeo’s 7-network is shown for each MS.

Using the activity regressors extracted based on equation (6) for each subject, we identified the correlated maps for each MS at the group level. As in the direct time course activity, each MS regressor was used independently in the GLM model. The results for the activity regressors are shown in figure 6 for MS-A, MS-B, MS-C, and MS-D.

### 3.4. Spatial maps of MS transition regressors

**Figure 7.**
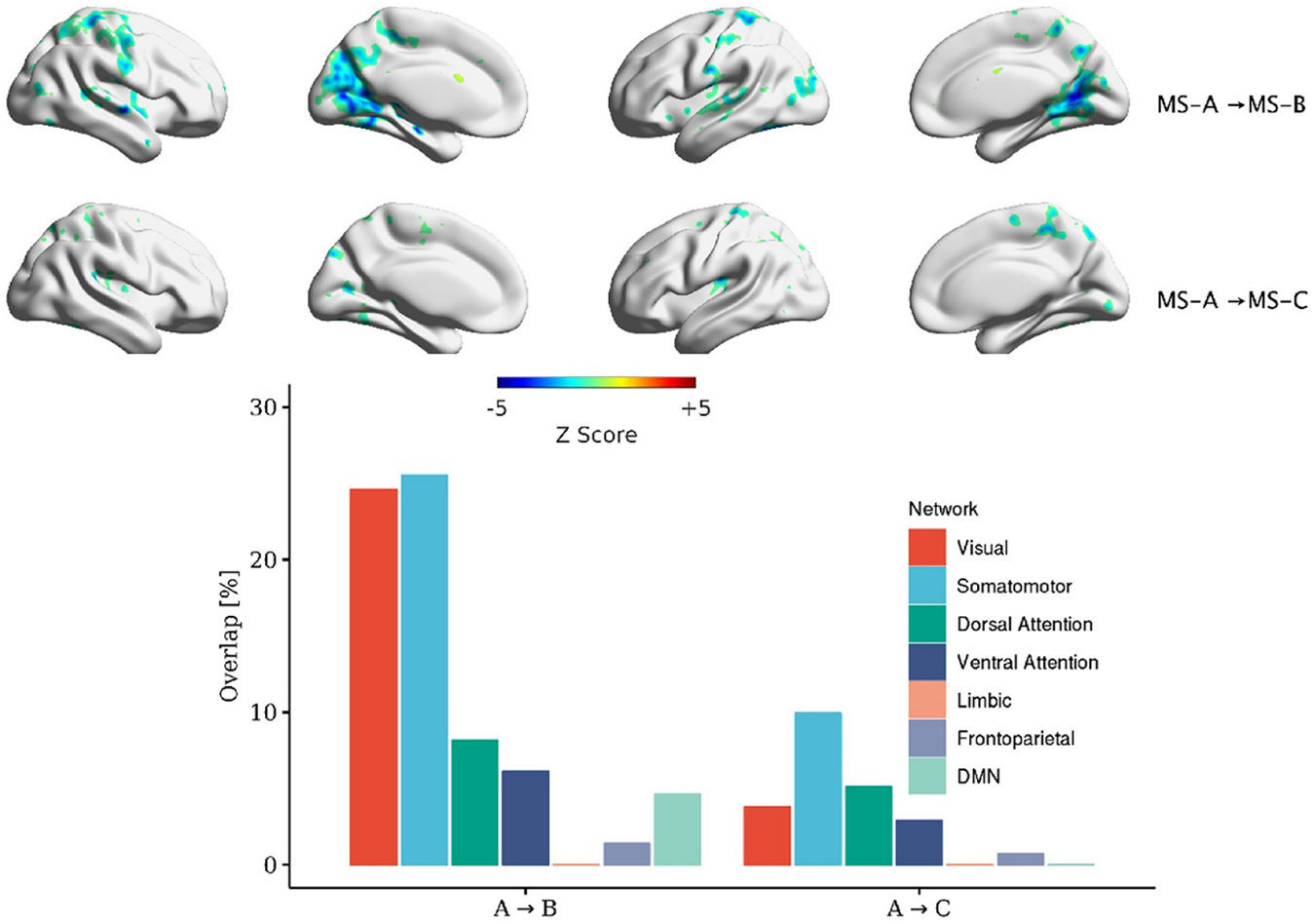
Significant clusters for transitions out of MS-A to other MSs. Clustering was performed at *p* < 0.005 and corrected at *p* < 0.05. The percentage of overlap with each of Yeo’s 7-network is shown for each transition. It should be noted that there were no significant clusters for the A→D transition.

**Figure 8.**
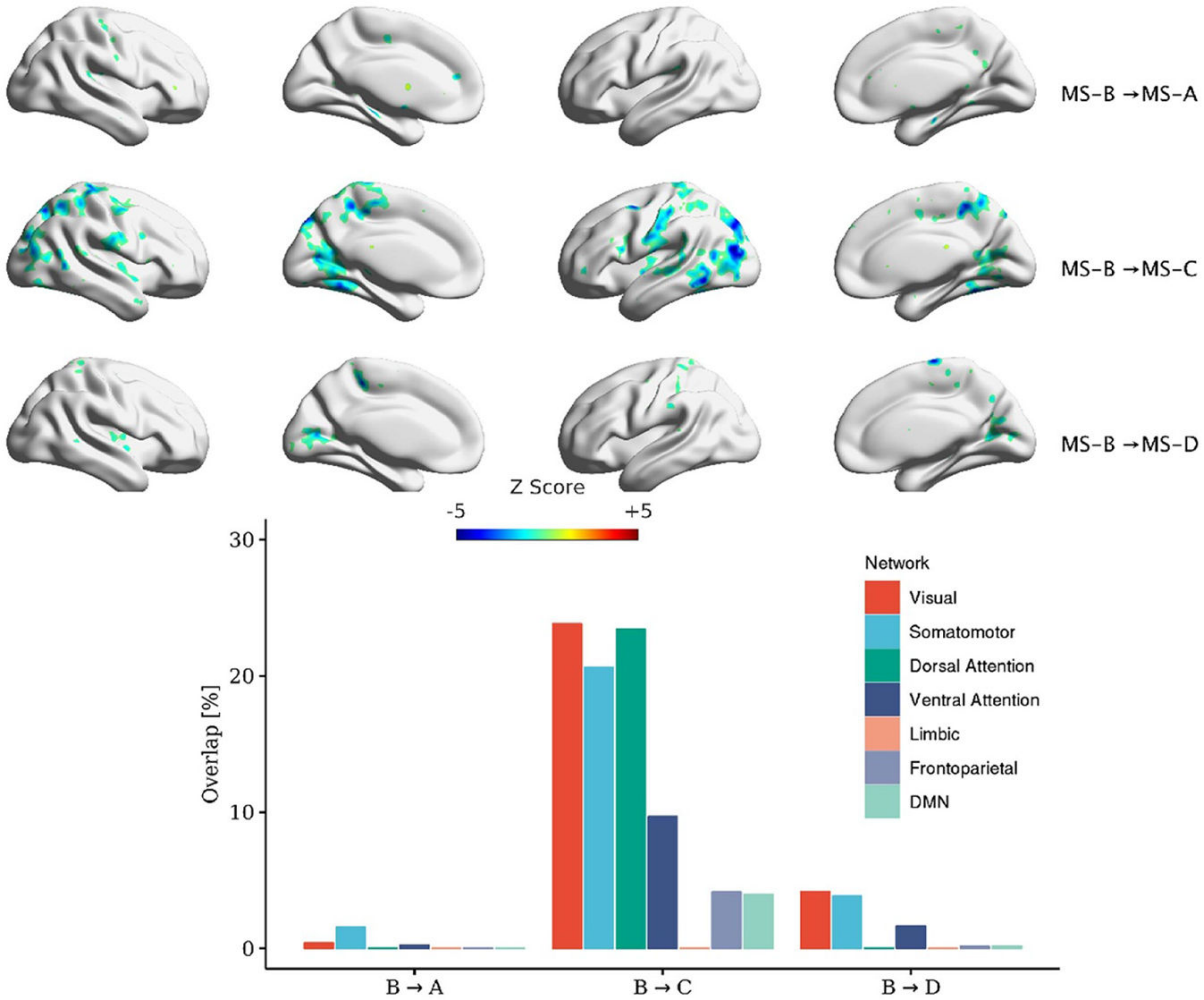
Significant clusters for transitions out of MS-B to other MSs. Clustering was performed at *p* < 0.005 and corrected at *p* < 0.05. The percentage of overlap with each of Yeo’s 7-network is shown for each transition.

**Figure 9.**
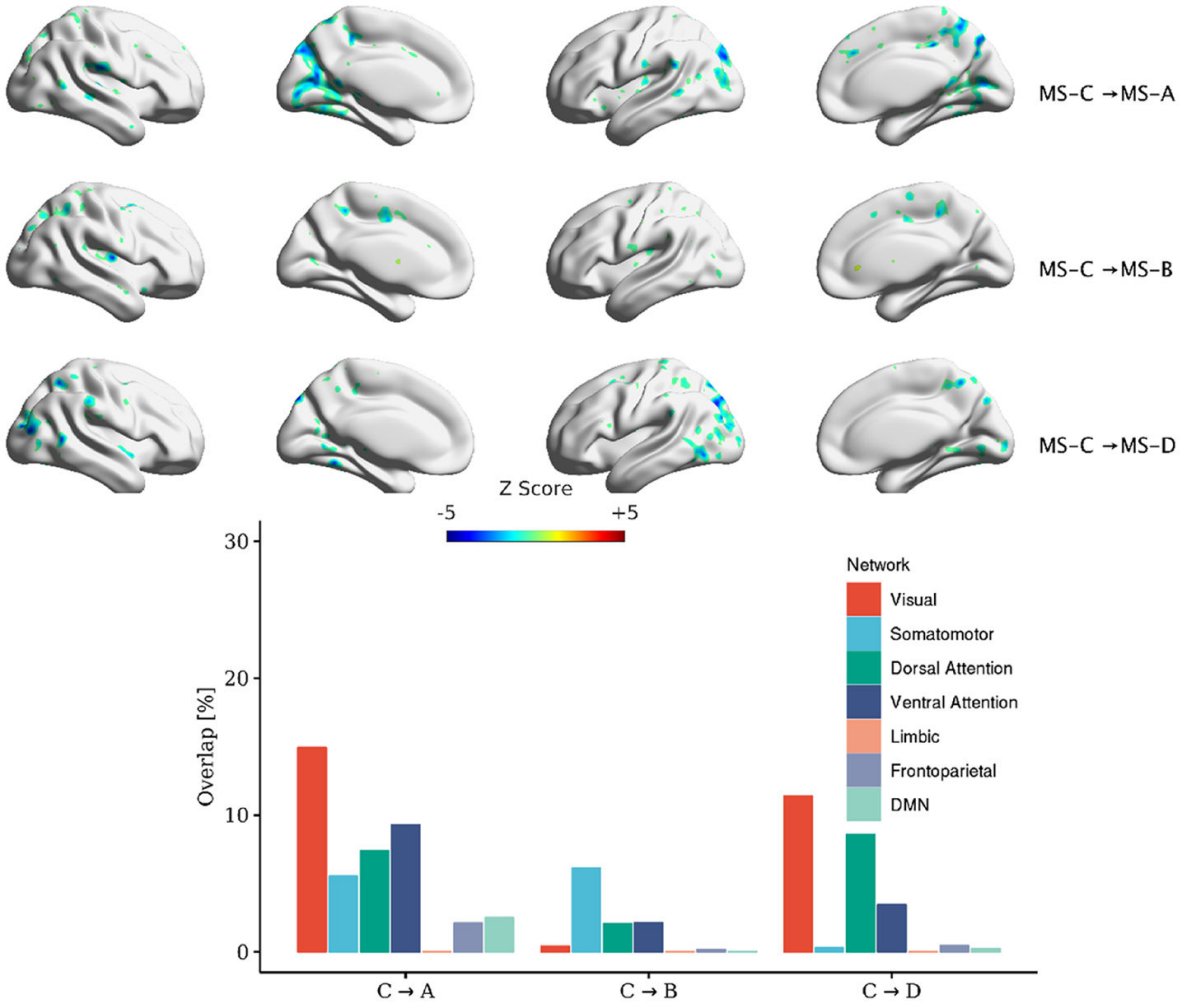
Significant clusters for transitions out of MS-C to other MSs. Clustering was performed at *p* < 0.005 and corrected at *p* < 0.05. The percentage of overlap with each of Yeo’s 7-network is shown for each transition.

**Figure 10.**
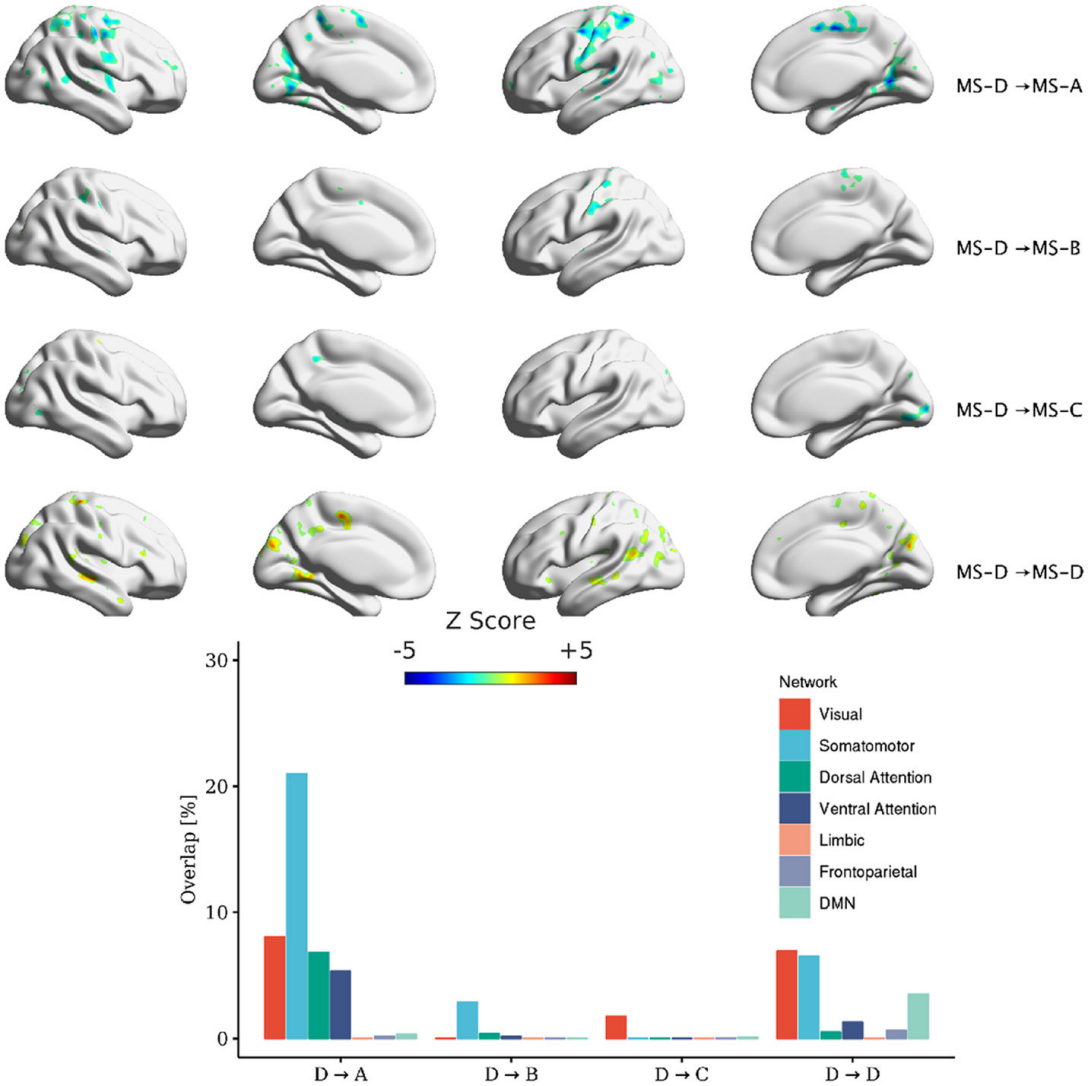
Significant clusters for transitions out of MS-D to other MSs. Clustering was performed at *p* < 0.005 and corrected at *p* < 0.05. The percentage of overlap with each of Yeo’s 7-network is shown for each transition.

MS lasts on average for ~50 ms before switching to another microstate. Using the transition regressors (equation (7)), we ran a GLM using 16 transition regressors and identified the correlation maps (12 transitions among four MSs and four regressors of self-transitions). Figures 7-10 depict the correlated map with significant clusters for each MS (if present). It should be noted that ${\text{TS}}^{A\to D}$ and three within microstate-transitions (${\text{TS}}^{A\to A}$, ${\text{TS}}^{B\to B}$, ${\text{TS}}^{C\to C}$) did not yield any significant clusters. All transitions have corresponding significant association except for ${\text{TS}}^{A\to D}$. Also, figures show different maps for symmetrical transitions (e.g. ${\text{TS}}^{A\to B}$ and ${\text{TS}}^{B\to A}$).

### 3.5. ML for modeling MS dynamics and BOLD signals

**Figure 11.**
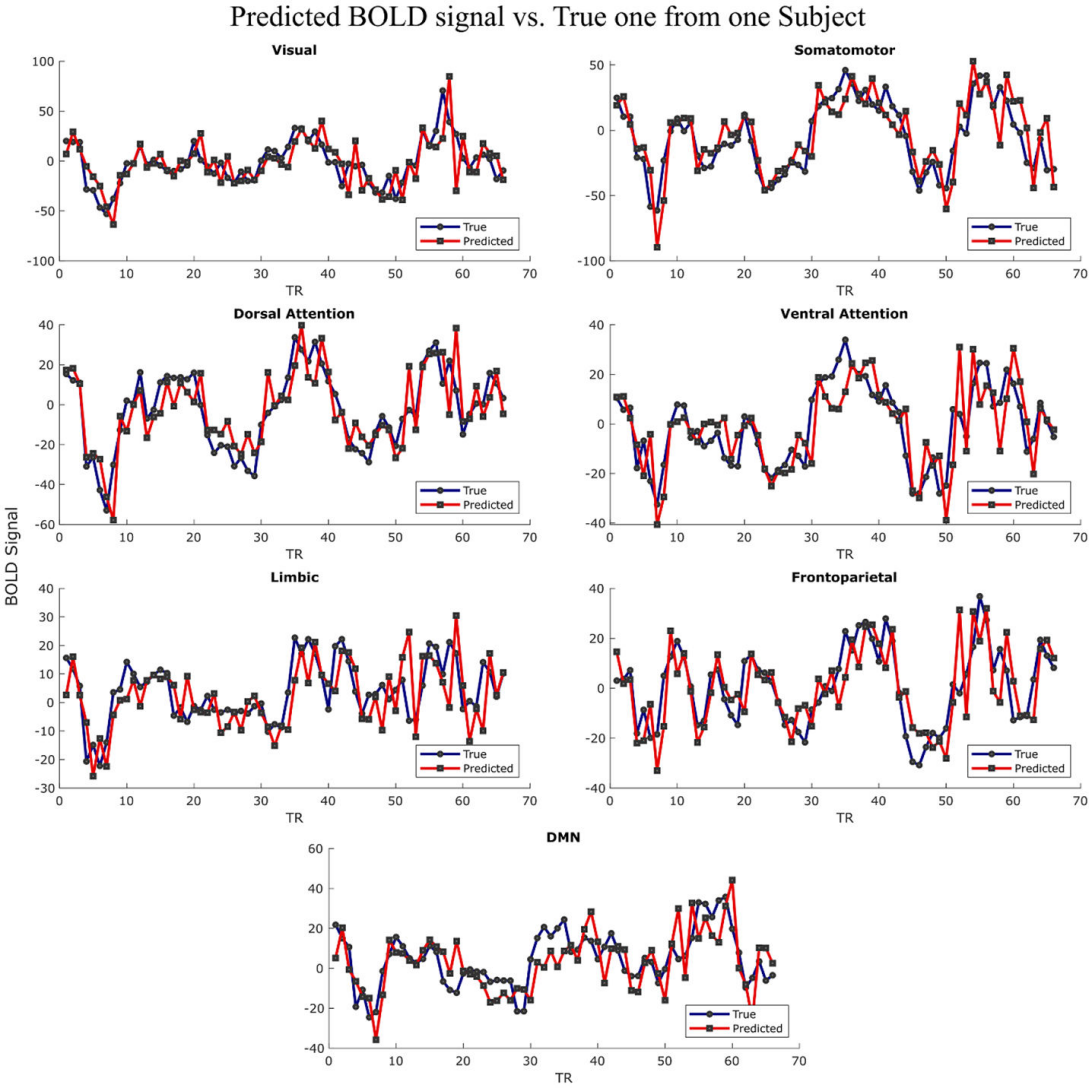
An example of the predicted BOLD signal versus true BOLD signal using mTDNN architecture for one step (1 TR).

**Figure 12.**
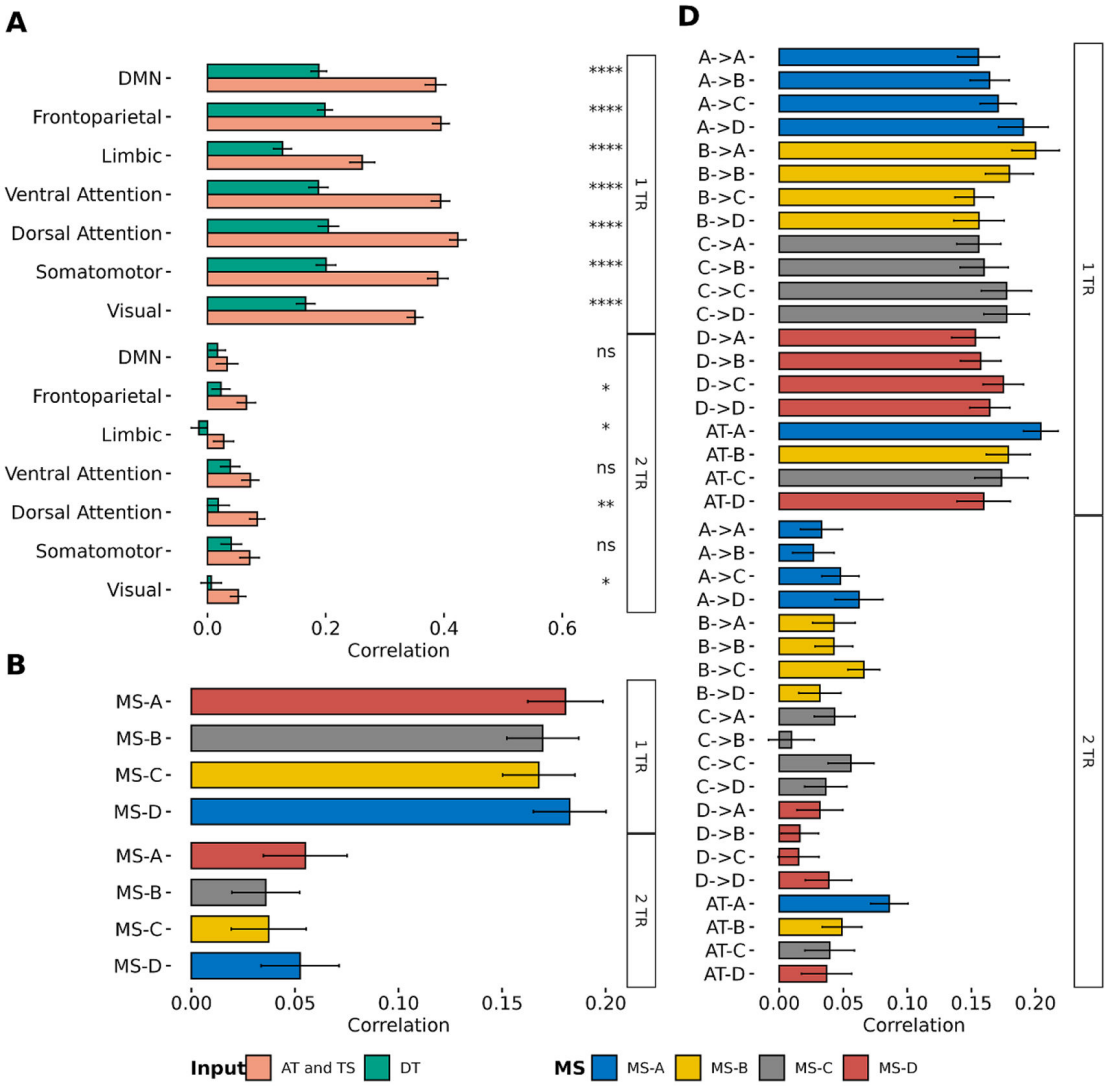
The average correlation between predicted and actual values for one and two steps ahead (1TR and 2TRs) using 40 HC subjects. Error bars represent the standard error for the correlation values. Figure (A) shows the correlation between the actual and the predicted BOLD signal for using either MS DT or MS AT and TS as inputs. Statistical difference between the two correlation values was conducted using the Wilcoxon test (**: *p* ⩽ 0.01, ****: *p* < 0.0001). Figure (B) shows the performance of predicting MS DT using the BOLD signal from 7-Network Yeo’s atlas. Figure (C) shows the performance of predicting MS AT and TS information using the BOLD signal from 7-network Yeo’s atlas.

GLM analysis revealed the spatial maps of each MS regressor with large distribution for MS dynamics. To further quantify the association between BOLD and MS regressors, mTDNN was adopted to model the relationship between MS and the BOLD signal. First, mTDNN was used to predict BOLD signals from Yeo’s 7-Network atlas) using either MS DT (input: 4 × DT regressors; output: 7 × BOLD signals) as input or MS dynamics (Input: 4 × AT regressors and 16 × TS regressors; output: 7 × BOLD signals). Second, the BOLD signal was used to predict the MS DT regressors (input: 7 × BOLD signals; output: 4 × DT regressors). Finally, the BOLD signal was used to predict MS dynamics regressors (Input: 7 × BOLD; output: 4 × AT regressors + 16 × TS regressors). In each experiment, mTDNN was used to predict one and two steps ahead (1 TR and 2 TRs). Figure 11 shows an example of the true and predicted BOLD signal by using MS dynamics (AT and DT) as an input. Figure 12 shows the performance of mTDNN from 40 HC subjects for all prediction tasks.

## 4. Discussion

This manuscript examined whether the spatial maps of the MS direct time courses, activity, and pairwise transitions can be related to fMRI BOLD imaging. Canonical MS are reliable and consistent patterns that can be identified by spatial clustering of the EEG electrodes (figure 4). Herein, we aim at providing a plausible interpretation of the stable topographical activation (lasting for approximately 50 ms) of MS. Some investigators have questioned the assumption that MS represent coherent brain activation of specific RSN due to the rapid nature of MS dynamics [2, 10, 72] and emerging results of the modulated effect of EEG rhythms on MS presence [34, 35]. To investigate in-depth the relationship between MS information and BOLD signal, we designed three sets of regressors to capture MS information: (a) the direct time course of each microstate, which represents the spatial similarities between each EEG point and MS templates; (b) the switching activity from specific MSs to other MSs, which measures the extent of switching from one brain function to other states; and (c) the pairwise transitioning among MSs, which reveals more detailed information about dynamics among switching between brain functions. Overall, there was a significant association between MS activity and transitions with the BOLD signal across several brain areas. Based on an ML approach, we are able to connect the fCMs results for the 24 regressors and their spatial distribution (figure 5: 4 × DT regressors; figure 6: 4 × AT regressor; figure 7: 4 × TS MS-A regressor; figure 8: 4 × TS MS-B regressor; figure 9: 4 × TS MS-C regressor; figure 10: 4 × TS MS-D regressor) to activation patterns in fMRI.

### 4.1. Statistical analysis

#### 4.1.1. DT regressors

Using the canonical MS DT regressors, nine significant clusters survived multiple-comparison correction: two clusters for MS-A, two clusters for MS-C, and five clusters for MS-D (figures 5, S3 and table 1). The results indicate a limited association between MS DT and the BOLD signal, while they are replicating previous findings. More specifically, the identified brain regions for MS-C (left middle frontal gyrus and cuneus) and MS-D (bilateral temporal gyri, left precentral and supramarginal gyri, and right cuneus) replicate findings for these MSs reported by [10]. In addition, our results indicated that MS-A exhibits a negative association with bilateral lentiform gyri, while no brain regions were associated with MS-B. The number of voxels surviving our multiple-comparison correction was relatively smaller than reported activations by [10]. This discrepancy might be due to our use of strict physiological and motion correction for resting-state analysis (RETROICOR and ventricle white matter regression). Similarly, [1–40] Hz configuration results revealed a limited association between MS DT and BOLD signal with only two and four significant clusters for MS-C and MS-D, respectively (supplementary: figures S4 and S5).

#### 4.1.2. AT regressors

The results from DT regressors suggested the need to assess the association between MS dynamics (switching and transitions) and the BOLD signal. Thus, we first investigated the extent of association between MS activity per each MS and the BOLD signal (figure 6). In other words, we tested whether MS switching is linearly associated with a change in the BOLD signal. The results revealed a strong negative association between MS switching and BOLD signals for all MSs. To characterize the fCMs, we used the 7-network atlas [51] to compute the percentage of overlap with each network. Results showed that MS-A, MS-B, and MS-C considerably overlapped with the somatomotor, visual, dorsal attention, and ventral attention networks. For all MSs, there was limited overlap with the default mode network (DMN), Limbic, and Frontoparietal networks. To offer a complete discussion, we investigated using [1–40] Hz configuration to extract the activity regressors. In this case, the analysis revealed a similar strong activation pattern to the [2–20] Hz case (supplementary: figure S6). Thus, BOLD signals were associated with switching per MS.

#### 4.1.3. TS regressors

We also investigated the association between MS pairwise transitions (4 × regressors × 4 MSs) and the BOLD signal. Figures 7-10 depict the fCMs for the connections with the significant cluster for MS-A, MS-B, MS-C, and MS-D, respectively.

Interestingly, a similar negative association with the BOLD signal was found as in AT regressors. We also highlight the following findings: (a) TS regressors did not show significant fCMs for all self-transition of MS (e.g. MS-A→MS-A) except for MS-D; (b) MS-D→MS-D is the only connection that showed a consistent positive association with the BOLD signal; and (c) the correlated maps of symmetrical transitions (e.g. MS-A→MS-B and MS-B→MS-A) were different. Additionally, we conducted the same analysis using [1–40] Hz frequency range to extract MS, which revealed similar fCMs found in the [2–20] Hz case (See figures S7-S10 in the supplementary).

### 4.2. ML analysis

The GLM analysis revealed the fCMs of each regressor and suggested that MS are associated with transitions and switching among brain networks. However, GLM analysis revealed only the linear relationship between MS information and BOLD signals. Thus, we adopted mTDNN to assess the non-linear association between MS and the BOLD signal. First, we showed for the first time that the BOLD signal could be predicted directly from MS information and vice versa. Several works attempted to model EEG directly from fMRI [49, 50, 73] to benefit from the portability and cost of using EEG only. Herein, we relied on using MS information to predict the BOLD signal. Unlike traditional EEG features, MS are broadband and multichannel representation of the brain neural activity. The proposed modeling, mTDNN, connected the previous information of MS/BOLD signals to predict the BOLD signal/MS information. mTDNN simultaneously identified the shared weights linking both modalities, which offers whole-brain modeling of two modalities. The results from mTDNN confirmed that MS activity and transition information are more associated with the BOLD signal than the direct time courses (figure 12(A)).

On the other hand, it showed that the BOLD signal could be used to predict from MS DT regressors (figure 12(B)) and MS AT and DT regressors (figure 12(D)). Although the association between MS DT and the BOLD signals was limited, as shown in the GLM analysis, mTDNN revealed a non-linear relationship between MS DT and the BOLD signal. In all cases, mTDNN performance degraded for predicting two-time points (two TRs) compared to a one-time point (figures 12(A)-(C)), suggesting that the mathematical model linking MS between BOLD is highly non-linear.

### 4.3. Plausible MS interpretation

In the light of the results of DT, AT, and TS regressors, the spatial distribution of fCMs suggests a stronger association among BOLD level and MS switching properties, than the direct time courses. That is, the observed time course or the presence of the MS label was less associated with the BOLD signal than the observed switching across MSs. To further elaborate on the interpretation of MS, we rely on evidence from the literature that suggests the following relationship among MS, EEG rhythms, and BOLD signal: (a) MS classes have been shown to be not only predominantly impacted by the strength and spatial distribution of alpha rhythm [34] but also modulate the MS metrics like occurrence, duration, and coverage [35]; (b) alpha rhythm has been shown to play a modularity role for the BOLD signal [36, 40] while showing negative association in the occipital regions [39, 42]; (c) alpha rhythm exhibits inhibitory behavior in the task-related brain regions [36-39, 74-76]; and (d) there is a lag in alpha rhythm changes when switching from task-negative to task-positive network reported in [77-79]. That is, based on the aforementioned points and results from AT, TS, and DT regressors, one could establish a plausible interpretation of MS presence using the tri-relationship among MS, alpha rhythm, and BOLD signal as a signature of switching from task-negative to task-positive networks. This interpretation explains the following observations: (a) the modularity effect of alpha rhythm on MS properties since alpha rhythm level change based on the task state and hence it changes MS properties; (b) the large spatial extent of the fCMs for AT and TS regressors, but not for DT ones; (c) the limited spatial overlap between DMN and fCMs of MS AT regressors since MS represents deactivation from task-negative regions to task-positive ones; (d) the limited special extent of fCMs for self-transitions since it means invoking the similar brain networks; (e) the fCMs were stable with irrespective to the MS frequency configuration; and (f) the short quasi-stabile MS topographies, which is attributed to the lag in the alpha power change when switching from task-negative to task-positive. Such interpretation of MS could explain some of the results in the literature as also pointed to by [34, 35]. For instance, the prominence of MS-C during resting-state reported by [80, 81] may be explained as a strong deactivation of task-negative regions and invoking other brain functions. This is also supported by the fact that there is an increase in the alpha activity associated with MS-C reported in [34] and that alpha rhythm inhibition was associated with activating task-related brain regions. Also, it could solve some inconsistencies in the literature, like the differences among the fCMS of MS-A and MS-B from [10] and the reported maps from [81] by interpreting the maps as the extent of inhabitation rather than specific brain function utilization. Although the provided interpretation is in line with several observations, other observations need further exploration. For example, we have shown that there is a lack of self-transition except for MS-D in both [2–20] and [1–40] Hz configurations with a positive association with the BOLD signal. Additionally, some pairwise transitions did not have a significant fCMs like MS-A→MS-D and MS-D→MS-C ([1–40] Hz). Additionally, the ML part supported our interpretation, but also suggested a non-linear relationship between MS DT and BOLD signals. It is not clear whether ML is capturing switching information manifested in the DT or the there is a direct non-linear relationship between MS DT and BOLD signals.

### 4.4. Methodological consideration

The length of the scanning time was 8 min. Longer scanning time might give better power for analyzing MS and BOLD signals. Also, the analysis used a 32-channel EEG recording to conduct our analysis. High-density EEG recording might also result in more robust results. Different numbers of TRs were used for different subjects due to censoring both EEG with bad interval points and fMRI with severe head motions. We relied on using Yeo’s atlas only to compute the overlap for each correlated map of each regressor template and extract BOLD signals for the ML part. Further analysis can be conducted to assess the stability of mTDNN weights and assess the non-linearity relationship. Finally, the results obtained here are based on resting-state fMRI analysis. Further analysis should be conducted using task-based fMRI to evaluate findings in this work.

## 5. Conclusion

EEG MSs have been used widely in the literature to characterize and understand brain networks’ neuronal activities, but their presence and temporal dynamics were still not well understood. The common assumption was that MS represents evoking specific brain networks. In this work, we further expand our understanding of the presence of MS by using simultaneous EEG-fMRI data from a large cohort of healthy subjects. We showed that MS direct time courses were limited in their association with the BOLD signal. On the contrary, MS activity and pairwise transitions revealed a strong negative association with the BOLD signals located mostly in the task-positive networks. We linked our findings with over two-decade of literature on MS, alpha rhythm, and the BOLD signal to suggest that MS represent the signature of switching from task-negative to task-positive networks, unlike the common assumption of single network associations. Our suggested interpretation of MS can explain many observations like short-time MS, their correlated maps, and importantly can explain conflicting findings in the literature. To further support our analysis, a multi-output RNN model confirmed the strong association between MS switching and BOLD signal more than the direct time courses of MS. Notably and importantly, we found that MS information could be used to predict BOLD signals and vice versa.

## Supplementary Material

Supplementary Material

## Figures and Tables

**Table 1.** Significant clusters using direct MS regressors. The coordinates are in MNI space.

MS	Cluster	Region	*X*	*Y*	*Z*	Voxel
A	C1	Right lentiform nucleus	−26	−2	2	231
A	C2	Left lentiform nucleus	28	−1	−1	187
C	C1	Left middle frontal gyrus	28	11	64	206
C	C2	Left cuneus	2	85	20	176
D	C1	Right cuneus	0	82	27	470
D	C2	Left supramarginal gyrus	50	58	26	361
D	C3	Left precentral gyrus	25	16	64	244
D	C4	Left middle temporal gyrus	60	40	−1	185
D	C5	Right superior temporal gyrus	−56	38	13	165

## Data Availability

Codes and summary results are available at https://github.com/obada-alzoubi/EEG_fMRI. The raw data generated during the current study are not publicly available for legal/ethical reasons but are available from the corresponding author on reasonable request.
