# Supplementary material for: Canonical EEG microstates transitions reflect switching among BOLD resting state networks and predict fMRI signal
Source: J Neural Eng. Author manuscript; Available in PMC 2024 Apr 11. (PMC11008726; doi:10.1088/1741-2552/ac4595)
Supplement: Supplementary Material [file NIHMS1980324-supplement-Supplementary_Material.pdf]

Supplementary materials to:

Canonical EEG Microstates Transitions Reflect Switching Among BOLD Resting State Networks and Predict fMRI Signal.

Canonical EEG Microstates Transitions Reflect Switching Among

BOLD Resting State Networks and Predict fMRI Signal.

Obada Al Zoubi[1,2,3], Ahmad Mayeli[1], Masaya Misaki[1#], Aki Tsuchiyagaito[1,4], Vadim Zotev[1],

Tulsa 1000 Investigators[1,3,5*], Hazem Refai[2], Martin Paulus[1], Jerzy Bodurka[†,1,6#]

[1]Laureate Institute for Brain Research, Tulsa, OK, United States
[2]Electrical and Computer Engineering, University of Oklahoma, Tulsa, OK, United States
[3]Harvard Medical School, Boston, USA
[4]Japan Society for the Promotion of Science, Tokyo, Japan
[5]Department of Community Medicine, Oxley Health Sciences, University of Tulsa, Tulsa, Oklahoma, United States
[6]Stephenson School of Biomedical Engineering, University of Oklahoma, Norman, OK, United States

[#]Corresponding author
[†]Deceased

Grid-search optimization of mTDNN architecture

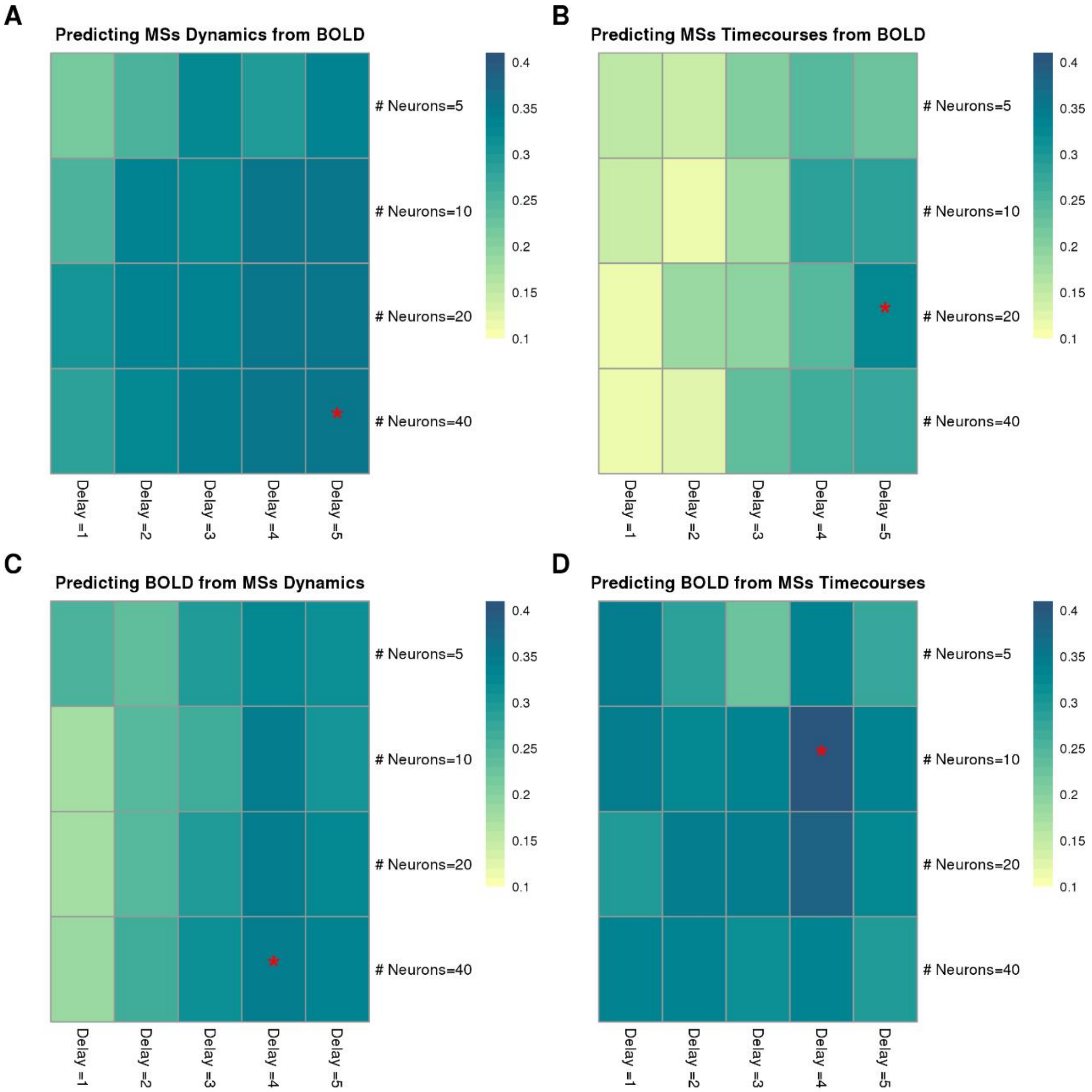

Figure S1: mTDNN architecture optimization results. Four experiments were tested including A) predicting MS dynamics from the BOLD signal; (B) predicting MS timecourses from the BOLD signal; (C) predicting BOLD signal from the microstates dynamics; and (D) predicting BOLD signal from MS timecourses. For each experiment, we averaged the Pearson's correlation among true and predicted outputs. The red astrict indicates the best performance. It should be noted that the analysis was conducted for the first 30 TRs of the signal

Time series cross-validation for predicting BOLD and MS properties

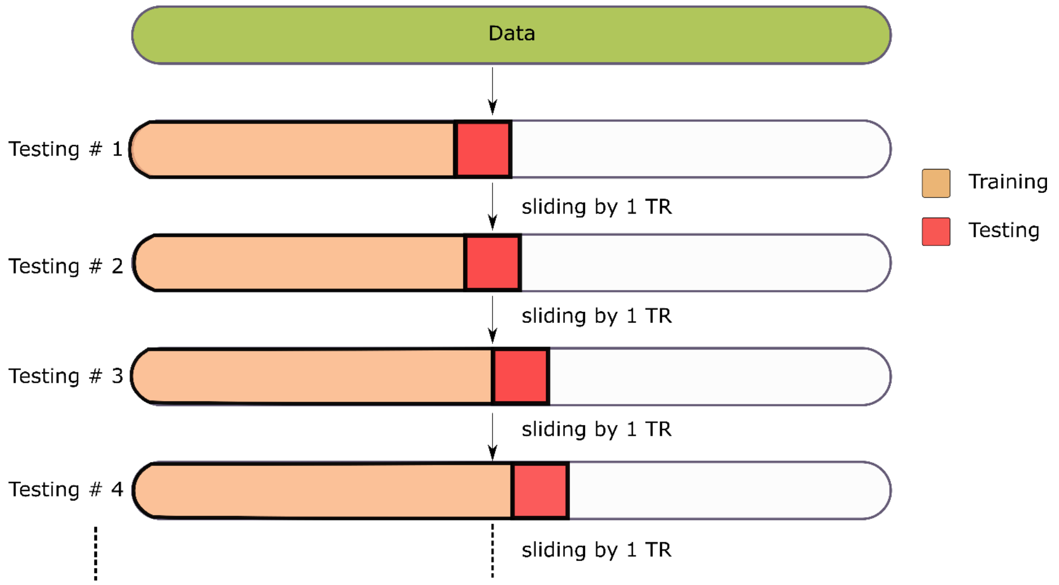

Figure S2: Time series cross-validation procedure.

EEG-ms Direct Time Courses Correlated Maps for 2-20 Hz filtering – thresholded

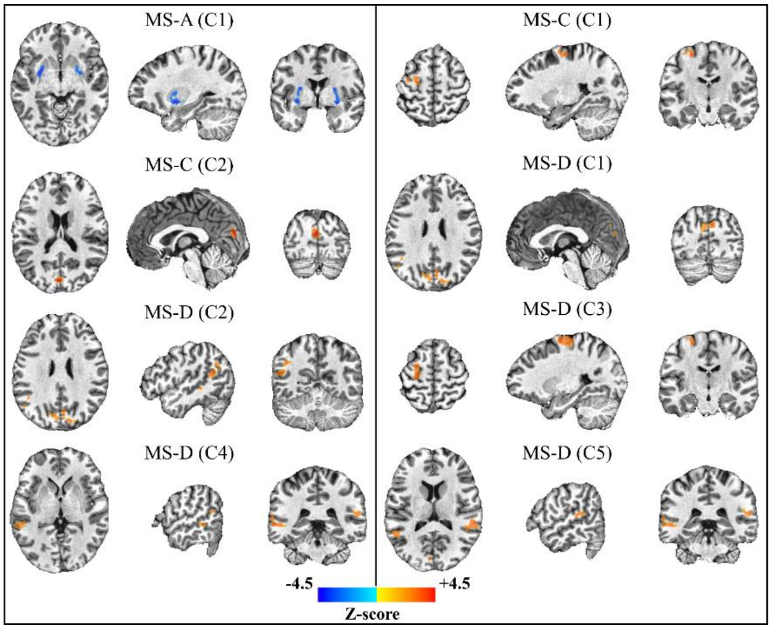

Figure S3: Significant clusters for MS-A, MS-C, and MS-D using direct time course regressors. Clustering was performed at p<0 .005 and corrected at p<0.05.

EEG-ms Direct Time Courses Correlated Maps for 1-40 Hz filtering

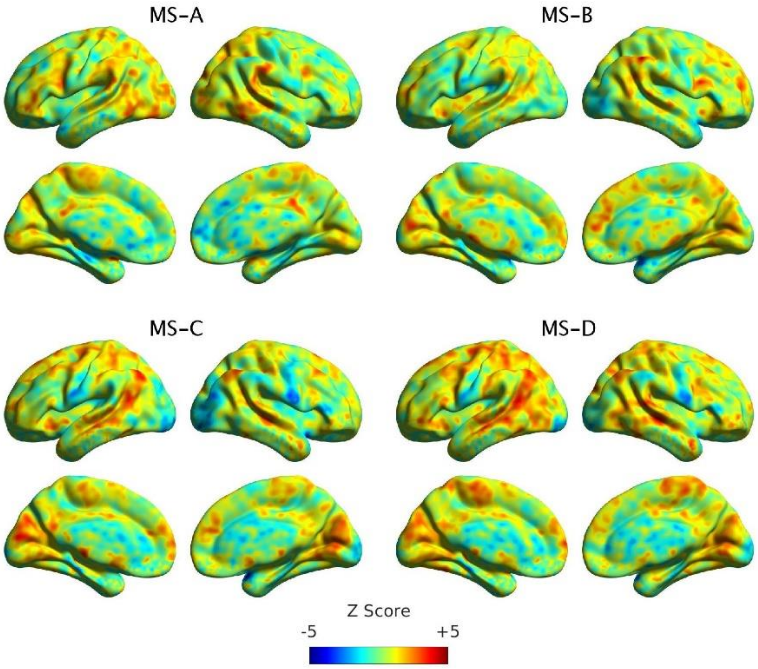

Figure S4: the un-thresholded maps of each the EEG-ms direct time regressors. No significant clusters were found for MS-A and MS-B.

EEG-ms Direct Time Courses Correlated Maps for 1-40 Hz filtering – thresholded

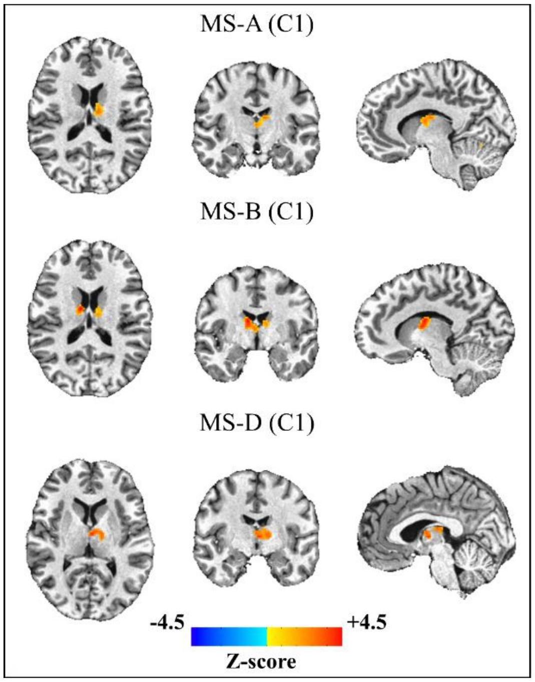

Figure S5: Significant clusters for MS-C and MS-D using direct time course regressors. Clustering was performed at p<0 .005 and corrected at p<0.05.

EEG-ms Activity per microstate Correlated Maps for 1-40 Hz filtering

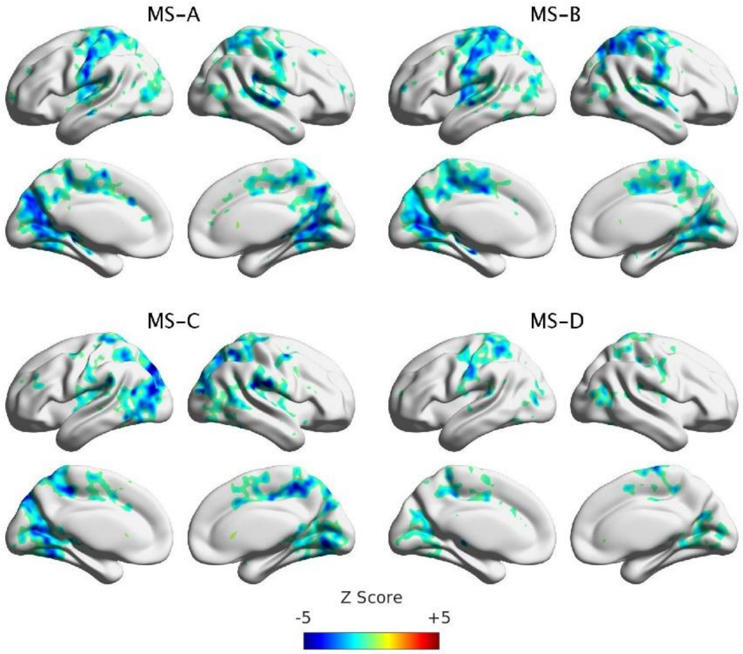

Figure S6: Significant clusters for MS-A, MS-B, MS-C, and MS-D using activity regressors. Clustering was performed at p<0 .005 and corrected at p<0.05.

EEG-ms pair-wise transition per microstate Correlated Maps for 1-40 Hz filtering

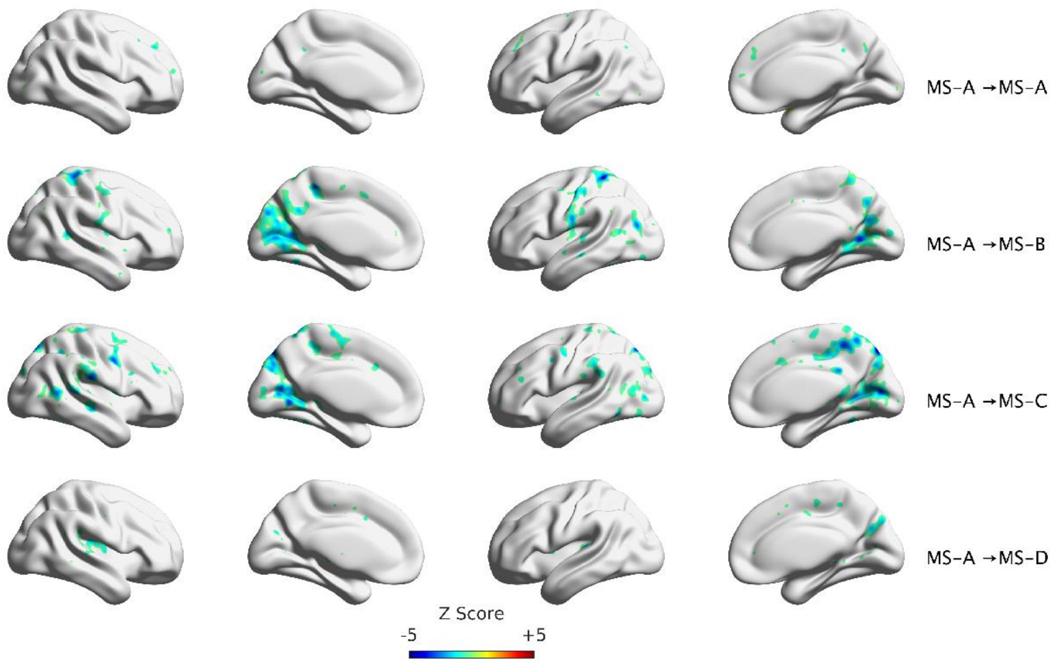

Figure S7: Significant clusters for transitions out of MS-A to other MSs. Clustering was performed at p<0 .005 and corrected at p<0.05.

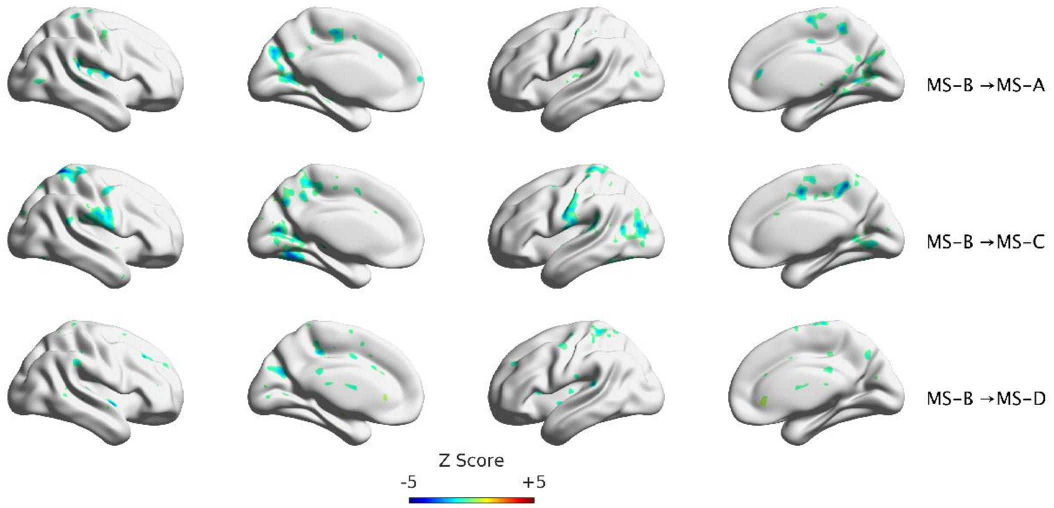

Figure S8: Significant clusters for transitions out of MS-B to other MSs. p<0 .005 and corrected at p<0.05.

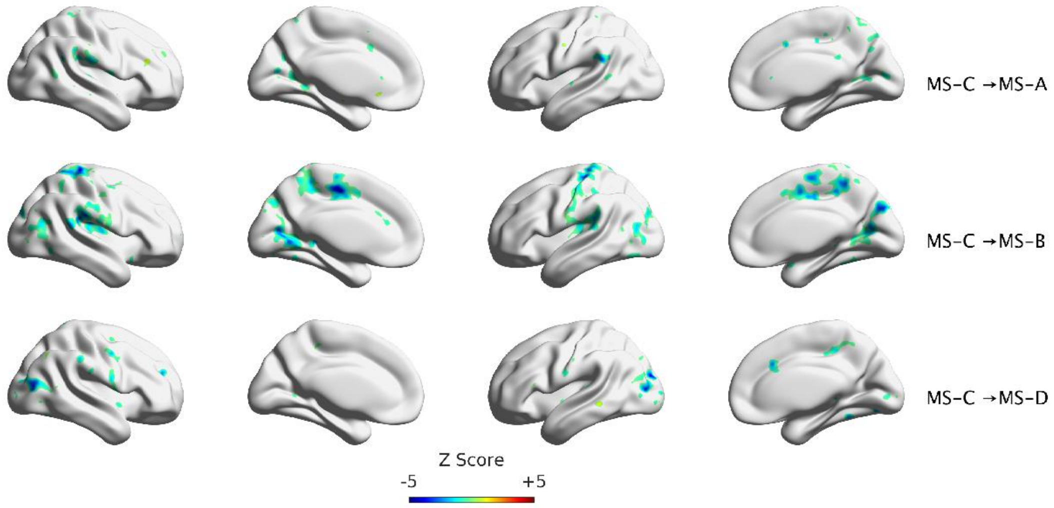

Figure S9: Significant clusters for transitions out of MS-C to other MSs. Clustering was performed at $p<0 .005$ and corrected at $p<0.05$.

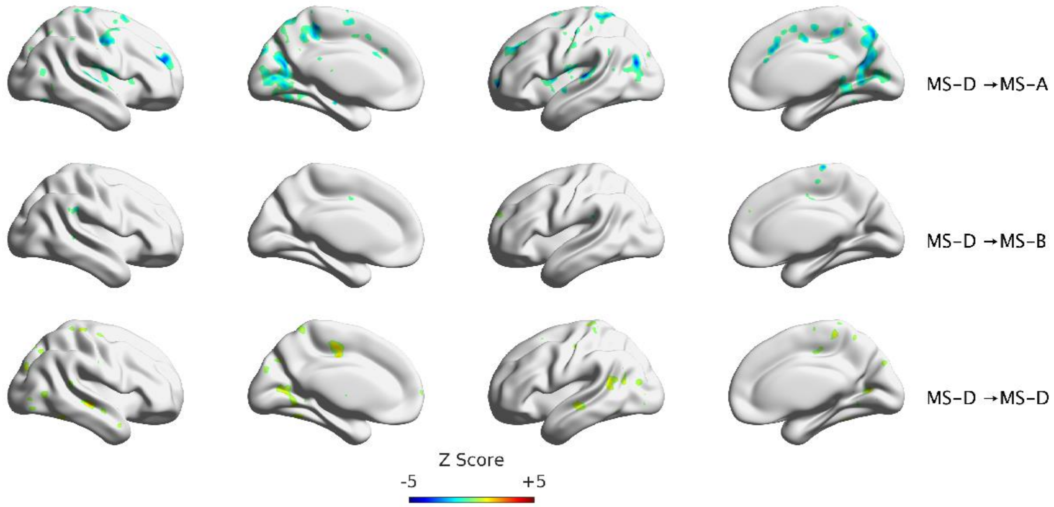

Figure S10: Significant clusters for transitions out of MS-D to other MSs. Clustering was performed at $p<0 .005$ and corrected at $p<0.05$.
